# The role of the histone H3 variant CENPA in prostate cancer

**DOI:** 10.1074/jbc.RA119.010080

**Published:** 2020-05-05

**Authors:** Anjan K. Saha, Rafael Contreras-Galindo, Yashar S. Niknafs, Matthew Iyer, Tingting Qin, Karthik Padmanabhan, Javed Siddiqui, Monica Palande, Claire Wang, Brian Qian, Elizabeth Ward, Tara Tang, Scott A. Tomlins, Scott D. Gitlin, Maureen A. Sartor, Gilbert S. Omenn, Arul M. Chinnaiyan, David M. Markovitz

**Affiliations:** 1 Medical Scientist Training Program, University of Michigan, Ann Arbor, Michigan, USA; 2 Program in Cancer Biology, University of Michigan, Ann Arbor, Michigan, USA; 3 Department of Internal Medicine, University of Michigan, Ann Arbor, Michigan, USA; 4 Program in Cellular and Molecular Biology, University of Michigan, Ann Arbor, Michigan, USA; 5 Michigan Center for Translational Pathology, University of Michigan, Ann Arbor, Michigan, USA; 6 Department of Computational Medicine and Bioinformatics, University of Michigan, Ann Arbor, Michigan, USA; 7 Department of Pathology, University of Michigan, Ann Arbor, Michigan, USA; 8 Department of Human Genetics, University of Michigan, Ann Arbor, Michigan, USA; 9 Howard Hughes Medical Institute, University of Michigan, Ann Arbor, Michigan, USA; 10 Program in Immunology, University of Michigan, Ann Arbor, Michigan, USA

**Keywords:** Centromere, prostate cancer, epigenetics, histone, gene expression, centromere protein A (CENPA), gene regulation, transcription, chromatin, cell proliferation, centromere

## Abstract

Overexpression of centromeric proteins has been identified in a number of human malignancies, but the functional and mechanistic contributions of these proteins to disease progression have not been characterized. The centromeric histone H3 variant centromere protein A (CENPA) is an epigenetic mark that determines centromere identity. Here, using an array of approaches, including RNA-sequencing and ChIP-sequencing analyses, immunohistochemistry-based tissue microarrays, and various cell biology assays, we demonstrate that CENPA is highly overexpressed in prostate cancer in both tissue and cell lines and that the level of CENPA expression correlates with the disease stage in a large cohort of patients. Gain-of-function and loss-of-function experiments confirmed that CENPA promotes prostate cancer cell line growth. The results from the integrated sequencing experiments suggested a previously unidentified function of CENPA as a transcriptional regulator that modulates expression of critical proliferation, cell-cycle, and centromere/kinetochore genes. Taken together, our findings show that CENPA overexpression is crucial to prostate cancer growth.

Centromeres are cellular structures that are necessary for the propagation of hereditary information (1, 2). Located centric to the ends of each chromosome, centromeres provide the structural foundation for kinetochores, multimeric complexes that serve as molecular interfaces between microtubule spindle fibers and individual chromatids during mitosis (1). The centromere–kinetochore–microtubule interaction facilitates separation of the sister chromatids as mitosis proceeds from metaphase to anaphase. Centromeres are thus essential to ensuring faithful segregation of chromosomes in actively dividing cells.

Efforts to study human centromeres have focused on the epigenetics that drive centromere assembly (3, 4). α-Satellite sequences that define centromere DNA are primarily occupied by a centromere-specific histone H3 variant known as CENPA (3, 5). CENPA is a functionally conserved ∼17-kDa molecule that forms a centromere-specific nucleosome with H2A, H2B, and H4 (6). Proper CENPA localization is an ubiquitin E3 ligase–dependent process requiring ubiquitination of lysine 124 for engagement with the CENPA-specific chaperone HJURP (7). HJURP subsequently facilitates the incorporation of newly synthesized CENPA into nucleosomes occupying replicated α-satellite DNA (8, 9). CENPA nucleosomes have a unique set of binding partners that facilitate proper genomic localization, including CENPB, CENPC, and the constitutive centromere-associated network (CCAN) that comprises the inner kinetochore (10, 11). The CCAN serves as a multimeric interface between the DNA-enveloped CENPA nucleosomes and the KNL-1–Mis12–Ndc80 complex network that comprises the outer kinetochore and directly interacts with the microtubule spindle fibers (12).

CENPA and its associated proteins therefore represent structural components that are essential to the integrity of cell division, and appropriate genomic localization of centromeric proteins is consequently a critical event in the cell cycle. Diseases of uncontrolled cell division, particularly cancer, are thus compelling to examine from the epigenetic perspective of centromere biology, primarily as it pertains to the key epigenetic mark CENPA. A number of studies have identified aberrant expression of centromeric/kinetochore proteins in cancers, where overexpression is predictive of survival and response to therapy, although their mechanistic contribution to cancer pathogenesis remains elusive (13–24). In the setting of ectopic constitutive overexpression, CENPA mislocalization in HeLa cells is independent of aberrant E3 ligase activity but rather demonstrates a reliance on the histone chaperone DAXX (25). Ectopic localization of endogenously overexpressed CENPA has also been shown in colon cancer cell lines (26). The phenotypic consequences of such mislocalization in malignancy have yet to be elucidated, although ectopic binding to sites marked by DNase hypersensitivity and CCCTC-binding factor (CTCF) transcription factor affinity has hinted at a potential role in regulating gene transcription (25, 26).

Here we report that CENPA is highly overexpressed in prostate cancer (PCA) and that disease progression correlates with CENPA expression within a large patient cohort. CENPA knockdown markedly decreases proliferation of prostate cancer cells but not that of benign prostate cells and increased expression of CENPA causes benign prostate epithelial cells to proliferate more rapidly. Interestingly, CENPA appears to affect proliferation of prostate cancer cells by acting as a transcriptional regulator that modulates expression of genes critical to proliferation, cell cycle progression, and centromere/kinetochore integrity in addition to its role in the centromere.

## Results

### Overexpression of centromeric factors in prostate cancer

**Figure 1.**
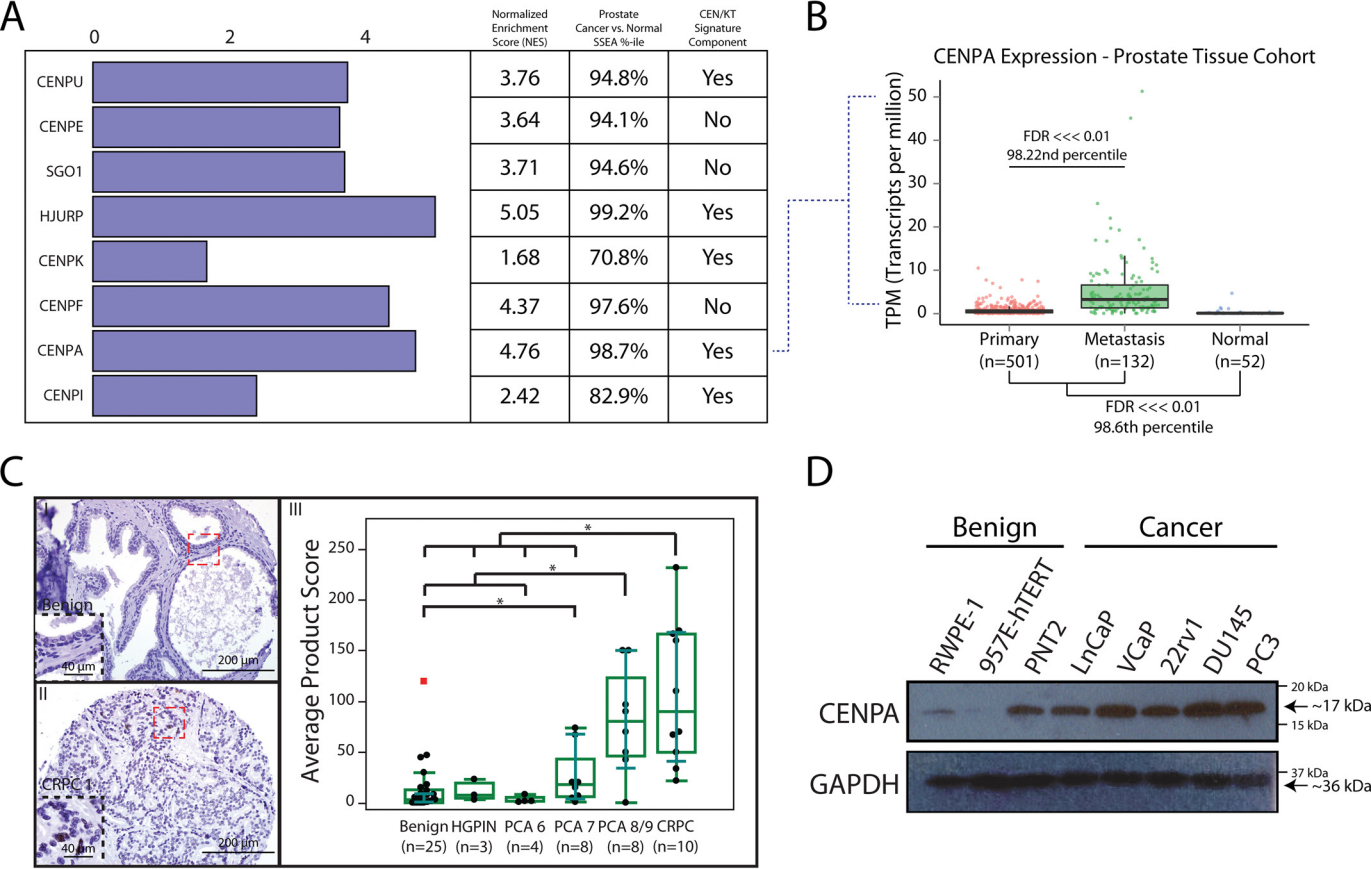
**Overexpression of CENPA in prostate cancer.** *A*, SSEA was used to query a catalogue of curated RNA-seq libraries (*n* = 685) for differentially expressed centromeric genes in the prostate tissue type cohort. Genes were selected based on associations identified in prior studies with cancer progression and were characterized by their inclusion in the previously described CEN/KT signature that negatively impacts therapy response and survival. *B*, focused SSEA on *CENPA* mRNA levels depicted as transcripts per million (*TPM*) in normal prostate (*n* = 52), primary prostate cancer (*n* = 501), and metastatic prostate cancer (*n* = 132) tissue. *C*, tissue microarray (*n* = 58 total tissues, *n* = 174 cores) of benign prostate (I), high-grade prostatic intraepithelial neoplasia (*HGPIN*), Gleason grade 6–9 prostate cancer (*PCA*), and castration-resistant prostate cancer (*CRPC*) (II) tissue stained for CENPA. *, *P* < 0.05. Staining was evaluated by assessing the most frequent pattern of intensity at 20× and the percentage of cells exhibiting that pattern (III). *D*, immunoblot for CENPA and GAPDH (loading control) in a panel of benign and malignant prostate cell lines. Note that PNT2, although benign, proliferates the most rapidly of all cell lines tested (see also Fig. S1*C*).

The significance of centromeres to cell division suggests that centromeric components may play important roles in development and in diseases involving cell division gone awry, particularly in cancer. Previous work identified a centromere-kinetochore (CEN/KT) signature that was associated with aggressive, treatment-refractory malignancy (16). We therefore profiled the transcriptomes of different types of malignancies across a compiled catalogue of publicly available RNA-sequencing (RNA-seq) databases (*n* = 10,848) (27). We found that *CENPA* is ubiquitously overexpressed in malignant tissue relative to respective normal counterparts (Fig. S1*A* and Table S1). These observations, combined with the well-characterized contributions of centromeric components like CENPA to cell division, suggested conducting a more focused interrogation of these components in cancers that display poor prognosis in the context of high proliferation indices. Prostate cancer is one such disease, where a high proliferation index is predictive of poor outcomes (28, 29). New treatment strategies are much needed for prostate cancer, which remains the most diagnosed malignancy in men and the second leading cause of cancer-related death in men (30). Although hormonal therapy and chemotherapeutic options are available, resistant metastatic disease and life-altering side effects, such as urinary incontinence and erectile dysfunction, are everlasting concerns (31). In view of the above considerations, we performed sample set enrichment analysis (SSEA) in the prostate tissue type cohort containing RNA-seq data from 685 tissue samples (27). Gene expression of numerous centromeric components exhibited strong enrichments in prostate cancer tissue relative to their normal counterparts (Fig. 1*A* and Table S2).

Our analysis corroborates on a significantly greater scale previous reports that characterize some of these components as part of the centromere-kinetochore (CEN/KT) signature that is strongly associated with poor disease outcomes (16). We selected *CENPA* from this panel of genes for further assessment, given its central role in centromere biology, importance for development, and highly conserved function, and found a significant increase in expression with disease progression (Fig. 1*B*) (32). This *in silico* finding was validated at the protein level through prostate tissue microarrays stained for CENPA, notably demonstrating marked overexpression of CENPA that increased with disease severity (*n* = 58 total tissues, *n* = 174 cores) (Fig. 1*C*). Importantly, receiver operator characteristic analysis of the CENPA-stained prostate tissue microarray produced an area under the curve of 0.89, orthogonally demonstrating a strong association between elevated CENPA expression and metastatic prostate cancer (Fig. S1*B*). Assessment of CENPA expression was also examined in cancer cell line models to determine feasibility for more focused molecular inquiry. We verified robust overexpression of CENPA in prostate cancer cell lines, as compared with benign prostatic epithelial lines (Fig. 1*D*). The PNT2 benign cell line was a notable exception, likely because of its rapid proliferation rate relative to other cell lines we tested (Fig. S1*C*). Taken together, CENPA is a functionally conserved, developmentally important factor abundant in prostate cancer tissue, as seen in a large number of patients and in prostate cancer cell lines, and an increase in its expression at the RNA and protein levels is highly correlated with more aggressive disease.

### CENPA is associated with cell division in prostate cancer

**Figure 2.**
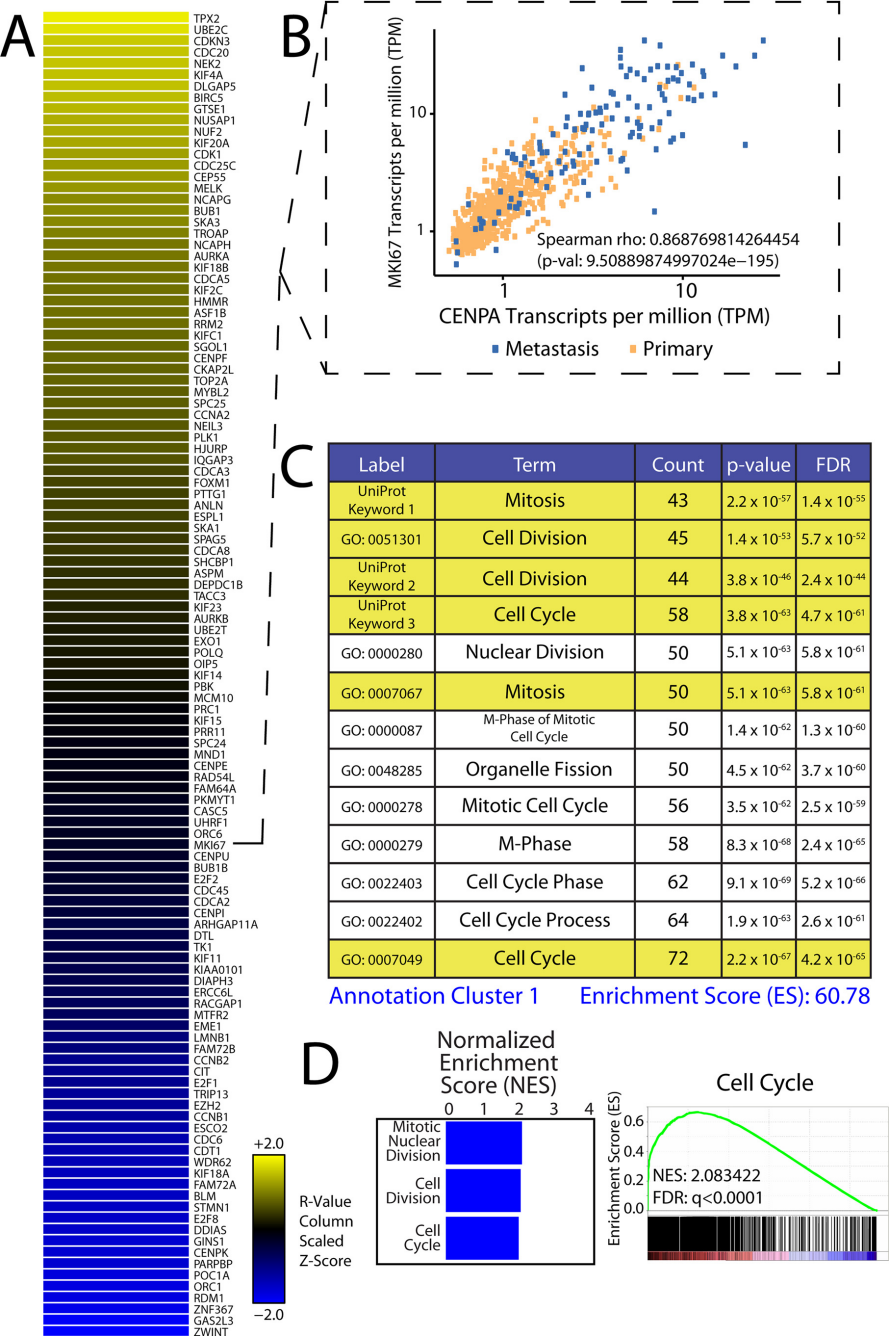
**Proliferation signature associated with CENPA.** *A*, *CENPA* mRNA levels from SSEA subjected to a transcriptome-wide correlation. The results were rank-ordered by the strength of correlation. The heat map depicts genes that performed at *r* ≥ 0.8. *B*, scatterplot depicting strong concordance between *CENPA* and the proliferation marker *MKI67*. Transcript abundance is depicted as transcripts per million (*TPM*). *C*, top 117 performers from transcriptome-wide correlation subjected to functional annotation analysis using the publicly available DAVID. Enriched biological concepts are rank-ordered by their FDR. *D*, independent GSEA of mitotic nuclear division, cell division, and cell cycle gene signatures conducted on transcriptome-wide correlation values preranked by the strength of correlation. The bar plot depicts enrichment scores from biological concepts designated along the *vertical axis* (*left*). Representative enrichment plot from the cell cycle gene signature is shown on the *right*.

The abundance of CENPA in prostate cancer raised the question of whether overexpression plays a functional role in disease pathogenesis and progression. We thus first conducted a comparative analysis of *CENPA* expression relative to the remaining transcriptome in prostate cancer to identify associations with biological concepts that could computationally guide functional assessments. Our efforts to profile transcriptomes in human cancer and normal tissue facilitates performing transcriptome-wide correlations against nominated genes of interest in a tissue-specific manner within a large catalogue of samples (*n* = 685). We thus correlated *CENPA* mRNA levels to the expression levels of all other protein coding elements (Data Set S1) to deconvolute its relative contribution to prostate cancer progression. *CENPA* expression tracks tightly with a number of previously identified prostate cancer pathogenesis factors including *CENPF*, *UBE2C*, and *EZH2* (Fig. 2*A*; Fig. S2, *C* and *D*; and Data Set S1). *MKI67* (gene encoding proliferation marker Ki67) also performed well in our analysis, further suggesting a role for *CENPA* in cellular proliferation (Fig. 2*B*). Of note, *CENPA* does not tightly correlate with *ACTB* (housekeeping gene), *AMACR* (prostate cancer biomarker), or *AR* (Fig. S2, *A* and *B*, and Data Set S1), suggesting a pathogenic process that is independent of androgen signaling, a pharmacologically relevant molecular pathway that is frequently targeted in prostate cancer treatment.

Strong associations with cellular proliferation genes and select pathogenesis factors independent of *AR* implicate *CENPA* as a contributor to a biological process that is involved in androgen refractory prostate cancer progression. In fact, we found that AR signaling actually represses *CENPA* expression in cell culture (Fig. S3*A*). We additionally used the Database for Annotation, Visualization, and Integrated Discovery (DAVID) to conduct ontology assessments on the highest-performing genes from our transcriptome-wide correlation against *CENPA* expression in prostate cancer (*r* > 0.8) (33). Our analysis revealed a correlation between *CENPA* gene expression and biological concept clusters that highlight centromeres, kinetochores, mitosis, and cell division (Fig. 2*C* and Fig. S3*B*). Concepts that include genes encoding components of the CCAN were additionally captured by our analysis. Of note, *CDC25C*, *CDCA5*, *TOP2A*, and *CENPU*, genes known to play roles in cellular proliferation, cell cycle progression, and centromere/kinetochore integrity, were included in these biological concepts. Preranked gene set enrichment analysis (GSEA) independently confirmed enrichments in gene signatures important for cell cycle, cell division, and mitosis (Fig. 2*D* and Fig. S3*C*). Taken together, *CENPA* expression is strongly linked to gene signatures that underlie processes that govern proliferation, cell cycle progression, and centromere/kinetochore integrity in prostate cancer.

**Figure 3.**
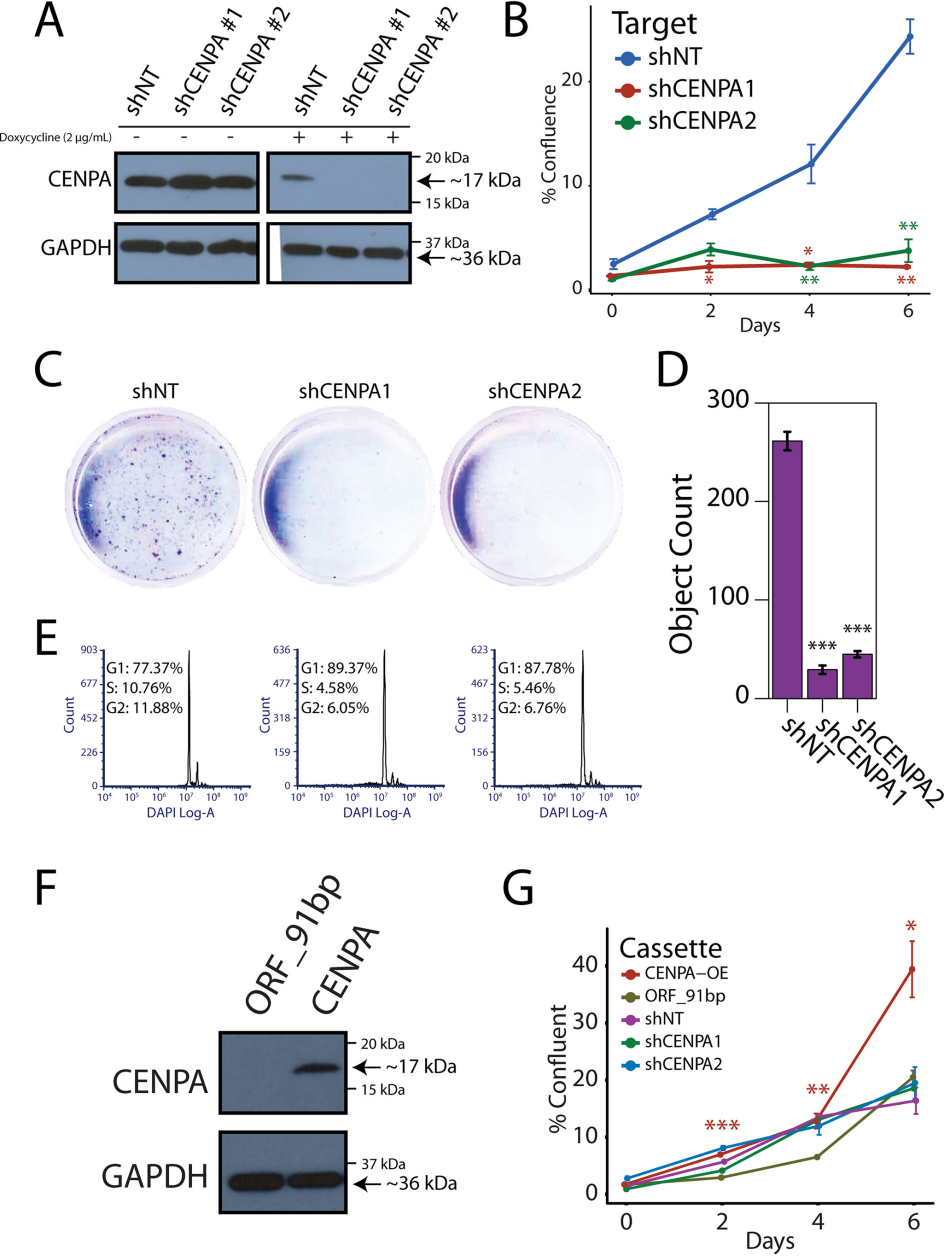
**Functional importance of CENPA in prostate cancer cells.** *A*, immunoblot for CENPA and GAPDH in 22Rv1 cells expressing a doxycycline-inducible vector encoding a nontargeted and two independent CENPA-targeted shRNAs. The cells were cultured with or without doxycycline at 2 µg/ml. *B*, growth curve depicting proliferation over 7 days following doxycycline induction in CENPA knockdown cell lines. *Error bars* represent the standard error of three biological replicates. *, *P* < 0.05; **, *P* < 0.01, compared with shNT for each condition via Student's *t* test. *C*, crystal violet cell proliferation assay conducted 7 days after doxycycline induction. *D*, quantification of the crystal violet colonies in *C*. *Error bars* represent the S.E. of three biological replicates. *E*, cell cycle analysis with 4′,6′-diamino-2-phenylindole (*DAPI*) in CENPA shRNA-depleted cells compared with shNT. *F*, immunoblot for CENPA and GAPDH in 957E-hTERT benign prostatic epithelial cells expressing a vector encoding a constitutively active CENPA construct (*CENPA-OE*). *G*, growth curve depicting proliferation over 7 days following CENPA overexpression or knockdown in 957E-hTERT cells. *Error bars* represent the standard error of three biological replicates. *, *P* < 0.05; **, *P* < 0.01; ***, *P* < 0.001, compared with CENPA-OE to ORF_91bp (vector control) via Student's *t* test.

### CENPA-dependent proliferation in prostate cancer

Significant association between *CENPA* and proliferation signatures is expected given the role *CENPA* plays in the structural integrity of the centromere. There is limited evidence, however, concerning CENPA function in human malignancy. We therefore performed loss-of- and gain-of-function experiments in cell lines stably expressing either doxycycline-inducible short hairpin RNAs against CENPA or EF1A promoter–driven full-length CENPA. Doxycycline administration at 2 μg/ml was sufficient to produce robust knockdown of CENPA after 72 h (Fig. 3*A* and Fig. S4, *A* and *B*). CENPA depletion led to a profound growth-inhibitory effect on 22Rv1, LnCaP, and DU145 prostate cancer cells (Fig. 3, *B–D*, and Fig. S4, *C* and *D*). CENPA depletion in prostate cancer cells results in an accumulation of cells in G_1_ that seem to be unable to progress through the cell cycle (Fig. 3*E* and Fig. S4, *E* and *F*). Conversely, overexpression of CENPA in the 957E-hTERT benign prostate epithelial cell line leads to a profound growth-promoting effect (Fig. 3, *F* and *G*). Interestingly, benign 957E-hTERT cells depleted of CENPA do not demonstrate significant proliferative changes, consistent with previous reports that nonmalignant cells can proliferate with low levels of CENPA (Fig. 3*G*) (34). Taken together, our data show that CENPA is an essential factor for progression through the cell cycle and that overexpression drives proliferation of prostate cancer cells.

**Figure 4.**
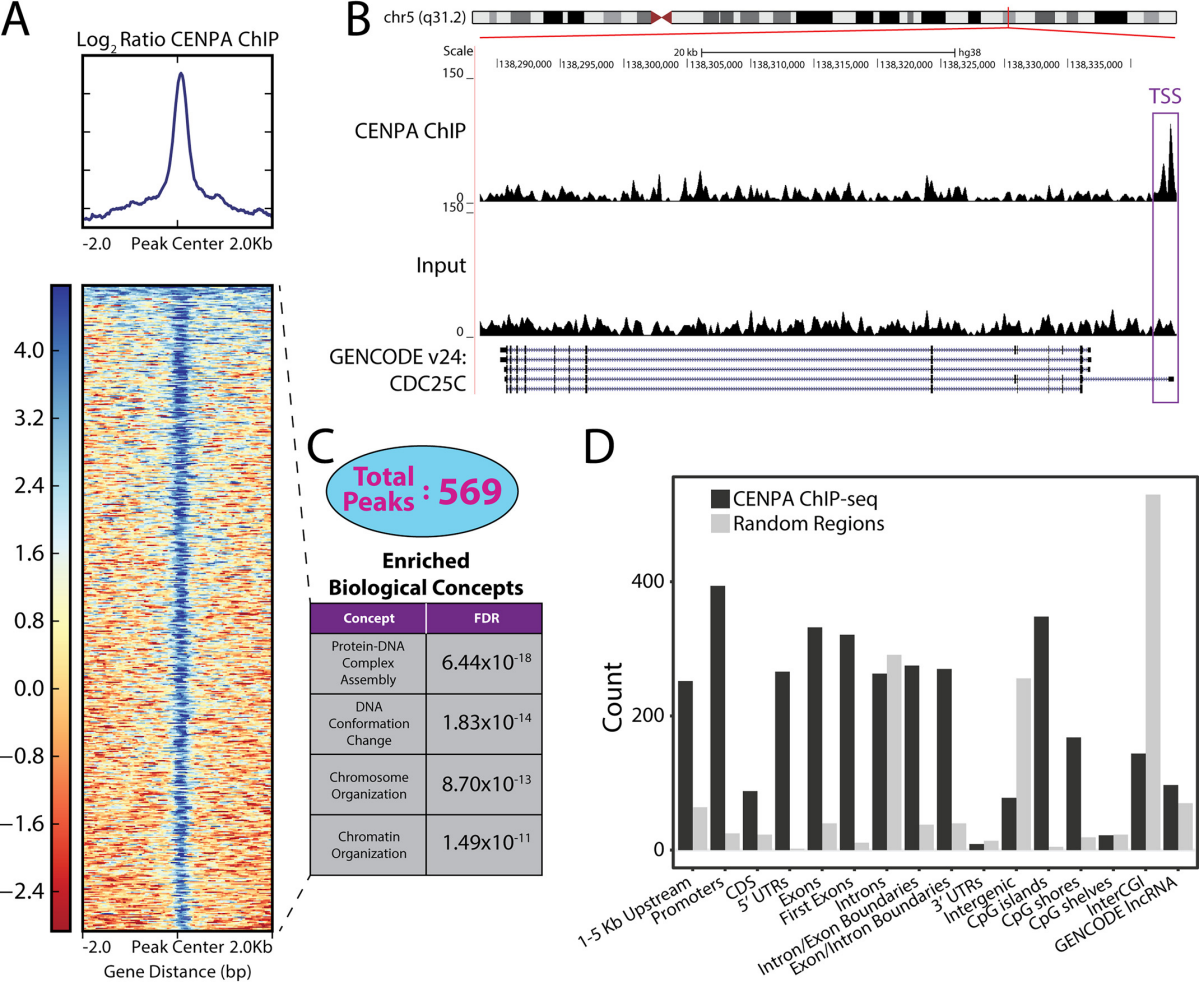
**Deposition of CENPA at regulatory regions across the genome in the VCaP prostate cancer cell line.** *A*, heat map across 4-kb windows of CENPA ChIP *versus* input signals centered at the CENPA peaks. *B*, UCSC Genome Browser illustration of CENPA binding to transcriptional start site (*TSS*) of the *CDC25C* gene on chromosome 5. *C*, 569 CENPA peaks were subjected to Gene Ontology assessment. Representative concepts are rank ordered by their FDR. *D*, CENPA peaks were annotated against 16 genomic regions relative to known genes. *CDS*, coding sequence; *CGI*, CpG Island. Peak abundance (*black bars*) was compared with abundance from random selection (*gray bars*) within each genomic region.

### Noncanonical genomic localization of CENPA

Given prior reports of ectopic deposition in the setting of CENPA overexpression and the marked overexpression of CENPA in prostate cancer, we performed native ChIP followed by sequencing to identify noncentromeric and potentially regulatory binding sites for CENPA in prostate cancer. As expected, four α-satellites were enriched relative to the IgG control antibodies using a PCR assay we previously devised that can distinguish chromosome-specific α-satellite DNA from any given centromere, verifying the validity of the CENPA ChIP (Fig. S5*A*) (35). CENPA-directed ChIP-seq identified 569 noncentromeric binding sites in the VCaP prostate cancer cell line within three experimental replicates (Fig. 4*A*; Fig. S5, *C* and *D*; and Data Set S2). One example of such a CENPA-binding site is present in the promoter region of *CDC25C* (Fig. 4*B*), a cell cycle phosphatase that is critical for progression through anaphase that was also identified in our comparative gene expression analysis described above (Fig. 2*A*). Intriguingly, the promoter of *CENPA* itself was also bound by CENPA, consistent with previously reported results, hinting that CENPA might regulate its own transcription (26). CENPA-directed ChIP was additionally conducted in the benign prostatic epithelial cell line 957E-hTERT to determine whether ectopic CENPA binding is a cancer-specific observation (Fig. S5*B*). CENPA enrichment over the four previously assessed α-satellites was significantly lower than that observed in the VCaP cell line, consistent with each cell line's respective CENPA abundance observed above (Fig. 1*D*). CENPA-directed ChIP-seq for the 957E-hTERT cell line was thus deferred.

**Figure 5.**
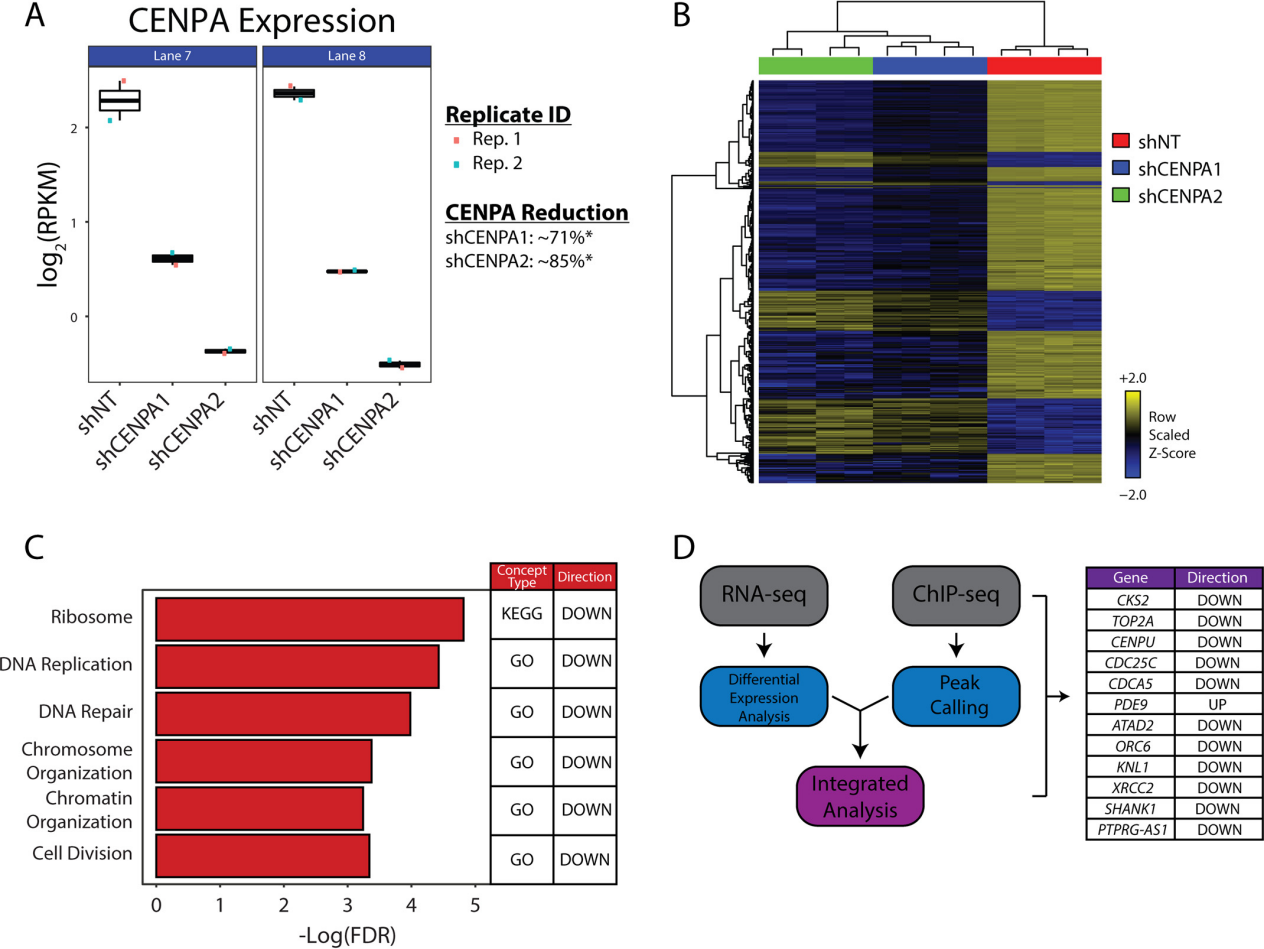
**Transcriptional profile of CENPA-depleted prostate cancer cells.** *A*, Jitterplot reflecting CENPA knockdown efficacy across all replicates (*Rep.*). CENPA mRNA levels are depicted as a logarithmic representation of reads per kilobase per million (*RPKM*) from all replicates. *B*, heat map representation of the 427 DEGs compared with a nontargeting shRNA to two independent CENPA-targeted shRNAs. Unsupervised hierarchical clustering was performed to group samples (*columns*) and genes (*rows*) by similarities in data structure. *C*, ontologic assessments conducted on the 427 DEGs using the RNAEnrich program. A subset of significant concepts from the analysis of CENPA-depleted cells are depicted. The Kyoto Encyclopedia of Genes and Genomes (*KEGG*) and Gene Ontology (GO) are databases that reflect ontologies representative of connected biological processes. *D*, transcriptional profile of CENPA-depleted 22Rv1 cells merged with CENPA ChIP-seq data from VCaP. The genes listed demonstrate both differential expression with CENPA depletion, as well as CENPA binding. The directionality of differential expression for each gene is depicted in the *right column*. Only genes that satisfy the absolute log fold change > 2, and FDR < 0.05 were considered for integrative analysis.

We next conducted a global assessment of CENPA-binding sites to obtain a functional taxonomy of CENPA-bound genes. Ontologic assessment of genes whose transcriptional start sites were in close proximity to CENPA-binding sites revealed enrichments in biological concepts that are involved with maintenance of nuclear architecture and organization, such as protein–DNA complex assembly (*p* = 6.44 × 10^−18^) and chromosome organization (*p* = 8.70 × 10^−13^) (Fig. 4*C*). Furthermore, binning CENPA-binding sites into categories corresponding to discreet locations within the human genome demonstrates a predilection toward binding regulatory elements such as promoters and CpG islands (Fig. 4*D*). Comparing the number of peaks present within any two genomic regions reveals significant overlap between loci considered to be regulatory areas (Fig. S5*E*). Taken together, we show that CENPA localizes to noncanonical genomic loci, with a predilection toward the regulatory elements of genes that control cellular proliferation.

### Gene regulation by CENPA

**Figure 6.**
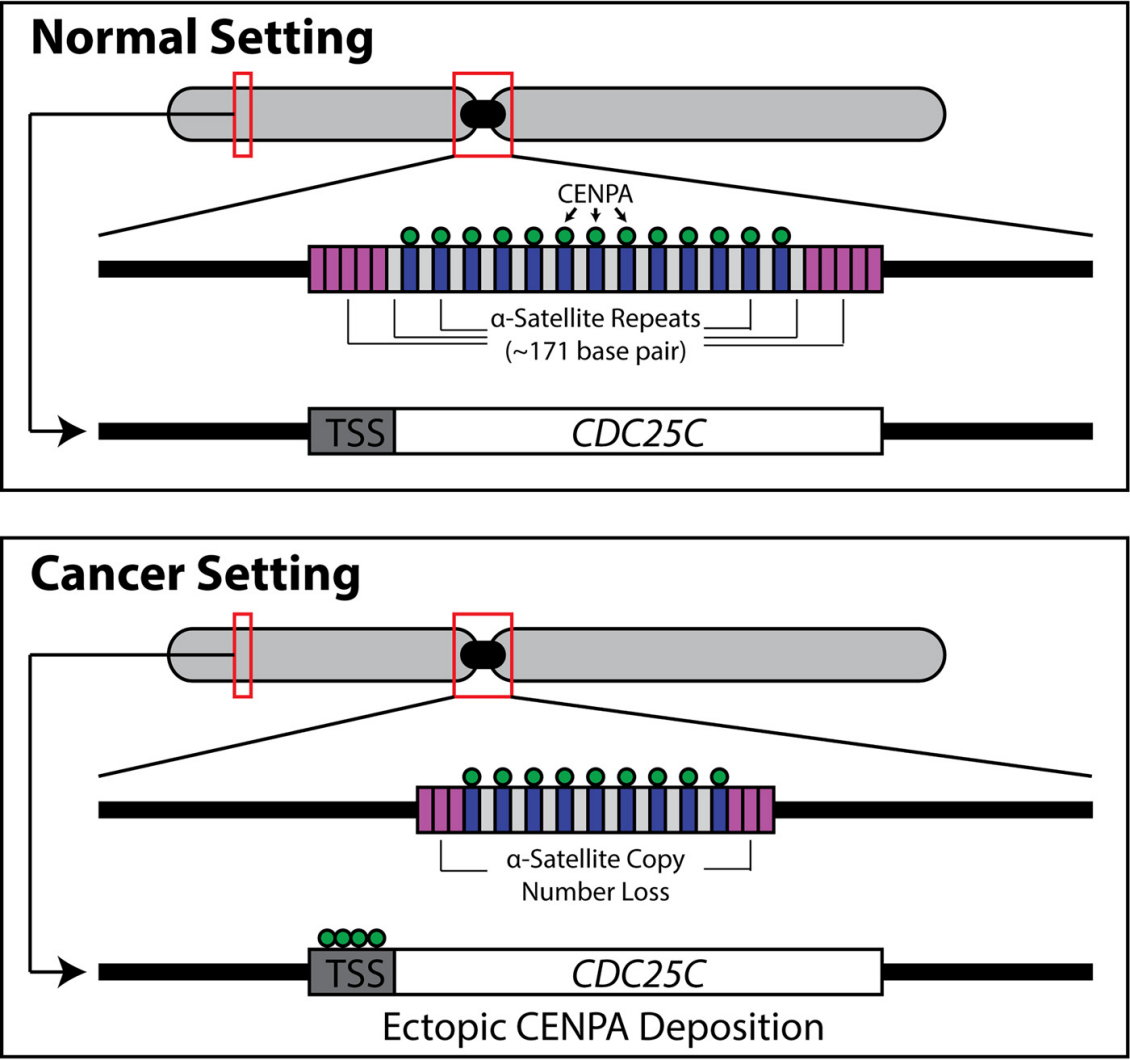
**Schematic depiction of centromeric molecular alterations in cancer.** Copy number alterations in the form of α-satellite deletions are observed across cancer types in both cell lines and tissue (50). CENPA, the H3 variant that traditionally occupies α-satellite DNA, ectopically binds gene regulatory elements, such as transcriptional start sites (*TSS*), of genes important for cell cycle progression, such as *CDC25C*, when overexpressed in cancer. Future studies are necessary to functionally link the ectopic localization to the α-satellite deletions.

Histone variants have been well-characterized as modulators of aberrant gene expression in cancer. H2A.Z.2, macroH2A, and H3.3 are well-documented as key contributors to malignant phenotypes in a number of cancer types (36–39). CENPA is a centromere-specific histone H3 variant overexpressed in cancer whose functional contributions to malignancy have remained elusive. CENPA localizing to regulatory elements outside of the centromere near genes involved in maintaining nuclear architecture and chromosome organization, however, presents the intriguing possibility that CENPA plays a direct role in gene regulation. We thus conducted RNA-seq on CENPA-depleted cell lines to evaluate whether removing CENPA drastically alters the gene expression profiles of genes bound by CENPA. Doxycycline administration at 2 μg/ml was sufficient to produce ∼71% (shCENPA1) and ∼85% (shCENPA2) knockdown of CENPA mRNA relative to the nontargeting control (shNT) after 72 h in the 22rv1 cell line (Fig. 5*A*). RNAi off-target effects were excluded by filtering genes that were either differentially expressed or dimensionally inconsistent between each independent CENPA-targeted shRNA (Fig. S6, *A* and *B*). The remaining 427 differentially expressed genes (DEGs) illustrated global transcriptional down-regulation in the setting of CENPA depletion (Fig. 5*B*, Fig. S6*C*, and Data Set S3). Indeed, when conducting ontologic assessments on the RNA-seq data set, we identified overlap between concepts bound by CENPA and concepts that are transcriptionally perturbed, specifically nuclear architecture and organization (Fig. 5*C*). Formal integrative analysis between the ChIP-seq and RNA-seq data (obtained from two different prostate cancer cell lines for technical reasons) additionally identified a number of genes essential to cellular proliferation and centromere/kinetochore integrity that were both bound by CENPA and differentially expressed in the setting of CENPA depletion (Fig. 5*D* and Data Set S4). *CDC25C*, *CDCA5*, *TOP2A*, and *CENPU*, genes whose expression levels were strongly correlated to CENPA expression in prostate cancer tissue, were all notably down-regulated with CENPA depletion, as well as bound by CENPA. Of note, cell division was also found to be an additional enriched biological concept within our RNA-seq data, again exhibiting similarity with earlier correlative findings in tissue. These findings collectively suggest that CENPA may be a regulator of transcription for genes important for proliferation and cell cycle progression in prostate cancer (Fig. 6), although additional integrative analysis involving the same cell line, as well as additional functional studies, will be required to confirm this hypothesis.

## Discussion

The centromeric histone H3 variant CENPA is overexpressed in cancer and has the propensity to localize to genomic loci that lie outside of the canonical centromere in the setting of overexpression. To date, however, the functional significance of the ectopic localization of CENPA in the biological setting of malignancy has been largely unexplored. We suggest for the first time that CENPA mislocalization may have functional consequences through an unexpected role as a regulator of gene expression in prostate cancer. *CENPA* is infrequently mutated and amplified in metastatic prostate cancer (40, 41). Given the relative genomic stability of the *CENPA* locus, the observed phenotypic aberrations that are the result of modulating CENPA expression are likely to be epigenetically driven. Although histone variants exhibiting aberrant biological properties in malignancy have been studied extensively, previous thought has been that CENPA's importance to cell division and proliferation is purely a function of its role as a structural node for the CCAN and KNL-1–Mis12–Ndc80 complex network. Although its role in the centromere is certainly vital, we now posit that ectopic deposition of CENPA in prostate cancer plays an additional role in modulating cell division by regulating critical proliferation, cell cycle, and centromere/kinetochore genes. These observations thus suggest an additional critical way in which this much-studied protein can affect cellular proliferation when overexpressed in the setting of cancer. Further experiments will be necessary to prove this hypothesis, however. This is especially true because despite many attempts using multiple different technologies, we were unable to obtain RNA-seq and ChIP-seq data from the same prostate cancer cell line.

Given the ubiquitous nature of CENPA overexpression in cancer, it is conceivable that ectopically bound CENPA driving proliferation through transcriptional regulation is a generalizable feature exhibited across numerous cancer types. Previous work shows that although centromeric/kinetochore proteins are infrequently mutated or amplified in cancer, coordinate overexpression of centromeric/kinetochore factors is a common characteristic identified in malignancy (13). Our findings suggest a potential epigenetic feedforward mechanism by which progressively increasing levels of CENPA drive gene expression of critical proliferation, cell cycle progression, and centromere/kinetochore factors that complement shifting mutational landscapes previously identified through cancer genomics approaches (42). Intriguingly, AR signaling, the most commonly targeted and mutated molecular signature in metastatic castration–resistant prostate cancer, was not captured by a CENPA-focused analysis, a finding that we further confirmed in tissue culture experiments. This phenomenon mirrors EZH2 expression changes in response to androgen stimulation, implicating CENPA as an additional epigenetic factor that may contribute to androgen-refractory progression (43). Of note, we observed that EZH2 expression indeed tracked tightly with CENPA expression in prostate cancer tissue (Fig. S2*D*).

Although we show here that prostate cancer tissue and cell lines overexpress CENPA, previous work suggests that occupation of only ∼4% of the α-satellite rich centromere is sufficient for producing functional centromeres (44). Indeed, benign prostatic epithelial cells do proliferate despite expressing low levels of CENPA and exhibiting reduced CENPA binding to α-satellite DNA. Loss-of-function experimentation yields pronounced growth inhibition in prostate cancer cell lines while producing no observable phenotype in a benign prostatic epithelial cell line. Moreover, overexpression of CENPA drives proliferation in benign prostatic epithelial cells. These findings are consistent with the concept that CENPA regulates cellular proliferation in the setting of overexpression and malignancy. Indeed, our data show that HJURP, the chaperone that directs CENPA to centromeric DNA, demonstrates strong concordance with CENPA expression in prostate cancer tissue. Work in HeLa cells additionally suggests that overexpressed CENPA is directed to gene regulatory elements through interaction with DAXX, a protein that has been previously been shown to be overexpressed in prostate cancer (45). It is thus conceivable that either HJURP or DAXX carries out a chaperone like function for CENPA in prostate cancer, although re-ChIP assays involving CENPA and HJURP together or CENPA and DAXX together would be necessary to prove the presence of such a mechanism.

It must be noted that previous studies that have evaluated transcriptional profiles in the setting of CENPA modulation have observed limited fluctuations in gene expression after loss-of- or gain-of-function experimentation (19, 25, 46). Although CENPA overexpression is rather ubiquitous across cancer types, it possible that differential gene expression seen in prostate cancer with CENPA modulation is tissue-specific. Evaluation of CENPA peaks for tissue-specific transcription factors, however, yielded statistically insignificant hits (data not shown). Additional cancer cell lines across numerous tissue types will require integrative analysis to better ascertain any tissue-specific contributions to our findings. Patient-derived primary cancer cell lines would additionally enhance the clinical applications of our work. It will be important to ensure that future integrative analyses maintain consistency in the choice of cell line to strengthen the findings presented in this study. Furthermore, given previous reports of genomic stress attributed to loss of CENPA, the altered gene expression subsequent to CENPA depletion that we report may be a phenotypic reflection of underlying mitotic defects that trigger cell cycle checkpoints, thus halting cell division and affecting the transcriptional program. Indeed, cell cycle arrest was noted in all cell lines depleted of CENPA. Evaluating the frequency of mitotic defects in the setting of CENPA knockdown will help determine whether gene expression anomalies secondary to CENPA modulation are in part due to the cellular response to genomic stress. However, given our findings that demonstrate CENPA occupancy at gene-regulatory elements across the genome, it is likely that the transcriptional program related to genomic stress is complementary to the transcriptional program activated by CENPA binding.

In conclusion, we show here that the much-studied centromeric histone H3 variant CENPA is markedly overexpressed in a wide panel of patients with prostate cancer, and overexpression correlates with disease progression. Further, CENPA is vital to the growth of prostate cancer cells and likely has a previously uncharacterized function as an epigenetic regulator of transcriptional activity involving genes important for proliferation, cell cycle progression, and centromere and kinetochore integrity in prostate cancer. CENPA overexpression, driven by as-yet uncharacterized oncogenic events, thus potentiates uncontrolled proliferation in prostate and perhaps other cancers. The ubiquitous nature of CENPA overexpression in other malignancies, in addition to prostate cancer, suggests that CENPA and factors downstream in its signaling pathway might be targeted for therapeutic purposes.

## Experimental procedures

### Cell lines and cell culture

LnCaP, 22Rv1, and DU145 prostate cancer cell lines were cultured in RPMI medium supplemented with 10% fetal bovine serum (FBS) (Atlanta Biologics) and 1% penicillin/streptomycin, as was the PNT2 prostatic epithelial cell line. VCaP and PC3 prostate cancer cell lines were cultured in Dulbecco's modified Eagle's medium supplemented with 10% FBS and 1% penicillin/streptomycin. The RWPE-1 and 957E-hTERT immortalized prostatic epithelial cell lines were cultured in keratinocyte serum-free medium supplemented with 0.05 mg/ml bovine pituitary extract and 5 ng/ml epidermal growth factor. All cell lines were grown at 37 °C in a 5% CO_2_ cell culture incubator, authenticated by short tandem repeat profiling for genotype validation at the University of Michigan Sequencing Core, and tested for *Mycoplasma* contamination.

### Tissue RNA-seq differential expression analysis

Analysis was performed on a compendium of 10,848 poly(A)^+^ RNA-seq libraries containing primary cancer tissue, normal tissue, and cancer cell lines from The Cancer Genome Atlas, the Michigan Center for Translational Pathology, and other public sources. SSEA was performed as previously described across all libraries to query CENPA expression in a cancer *versus* normal fashion (27). Further analysis restricted to the prostate tissue type cohort was conducted to determine CENPA expression levels at different stages of malignancy.

### Ontologic assessments

CENPA mRNA levels within the cancer cohort were subjected to transcriptome-wide correlation studies against all protein-coding genes. All genes satisfying the *r* > 0.8 criteria were included in a custom list for ontologic assessments. This list of genes was ranked by the Spearman rho coefficient and subject to pathway analysis. The DAVID tool performed ontologic assessments against Gene Ontology terms as well as UniProt concepts. Weighted, preranked GSEA was performed against MSigDB data sets.

### Tissue microarray

CENPA expression in prostatic epithelium was assessed by immunohistochemistry on a tissue microarray using a mouse monoclonal anti-CENPA antibody (MBL International). Benign prostate tissue, high-grade prostatic intraepithelial neoplasia, localized prostate cancer and metastatic castration–resistant prostate cancer tissues were spotted in triplicate on the core (*n* = 58 total tissues, *n* = 174 cores). Staining was evaluated by assessing the most frequent pattern of intensity at 20× in addition to the percentage of cells showing that pattern. A product score was subsequently calculated (intensity × percentage of cells with the pattern) for each core. The receiver operator characteristic curve was generated based on average product scores.

### Quantitative reverse transcriptase–PCR (qRT-PCR) assay

The All Prep DNA/RNA mini kit (Qiagen) was utilized to isolate RNA from cell lysates. RNase-free DNase (Qiagen) was used to eliminate contaminating genomic DNA. RNA was quantified by the NanoDrop 2000 (Thermo Fisher Scientific) and diluted to 25 ng/μl. The Step One Plus real-time PCR system (Applied Biosystems) was utilized for One-Step qRT-PCRs and Moloney murine leukemia virus reverse transcriptase (Promega) for reverse transcription. Gene-specific primers were designed and subsequently synthesized by IDT Technologies. A relative quantification method was used to analyze qRT-PCR data and subsequently presented as average fold change over an internal reference (as an internal reference, GAPDH was utilized). All of the primers used for quantitative PCR are detailed in Table S3. Three technical replicates were used in each assay, and all of the data shown were from three biological replicates.

### Lysates, antibodies, and immunoblotting

The cells were pelleted, dissolved in 4× Laemmli buffer, sonicated for 30 s, and immediately placed in ice. Whole-cell extracts were then heated for an additional 2 min at 95 °C and returned to ice. The samples were subsequently separated on 4–20% SDS-polyacrylamide gels (Bio-Rad) and transferred to polyvinylidene fluoride membranes via wet transfer at 80 V for 90 min. The membranes were then incubated in blocking buffer (PBS, 0.1% Tween, 5% nonfat dry milk) for 1 h at room temperature. Primary antibody incubations were conducted with indicated antibodies in blocking buffer at 4 °C overnight. Secondary antibody incubations were conducted the following day with species-appropriate horseradish peroxidase–conjugated secondary antibodies. The blots were developed using enhanced chemiluminescence substrate according to manufacturer's protocol (Millipore). Antibodies against CENPA (Abcam ab13939) and GAPDH (Abcam ab181602) were used as primary antibodies.

### Knockdown and overexpression studies

Stable knockdown of CENPA was achieved using the pTRIPZ Tet-On system (Dharmacon). Commercial glycerol stocks of bacteria propagating plasmids containing two different CENPA-directed shRNAs and a nontargeting shRNA were inoculated and cultured for 24 h. Plasmid DNA was subsequently isolated using a plasmid maxi kit (Qiagen) and sent to the University of Michigan Vector Core for lentiviral production. 22Rv1, DU145, and LnCaP cells were transduced with lentivirus in the presence of 8 μg/ml Polybrene. After 24 h, the cells were cultured in the presence of 2 μg/ml puromycin. Knockdown was achieved through culturing cells with doxycycline at a final concentration of 2 μg/ml. Doxycycline response was assessed by microscopy, immunoblot, and qRT-PCR. Stable CENPA overexpression was achieved using the pLV system driven by an EF1A promoter (VectorBuilder). Plasmids and lentivirus were prepared as described for the pTRIPZ system. 957E-hTERT cells were incubated with lentivirus in the presence of 8 μg/ml Polybrene. After 24 h, the cells were cultured in standard keratinocyte serum-free medium and subjected to FACS to select mCherry-positive cells by the University of Michigan Flow Cytometry Core. Overexpression was verified by qRT-PCR and immunoblot. Maps of all vectors are provided in the supporting information.

### R1881 treatment

To evaluate the effect of androgen signaling, the cells were cultured in medium containing 10% charcoal-treated FBS and treated with DMSO vehicle or with 1 or 10 nm R1881. After 24 and 48 h, RNA was isolated and qRT-PCR was performed as described above using FastStart SYBR Green Mastermix (Roche).

### Cell proliferation assays

The cells were seeded in T-25 flasks at 1 × 10^6^ cells/flask. The flasks were evaluated by microscopy 24 h following seeding to assess initial confluence. Growth curves were constructed by imaging flasks by microscopy, where the growth curves are generated from confluence measurements acquired from 6 fields/condition, using the NIS Elements microscope imaging software. Proliferation assay was performed for shRNA-mediated knockdown, overexpression, and basal growth experiments.

### Crystal violet assays

The cells were seeded in triplicate in 6-well plates at 1 × 10^4^ cells/well. The cells were given 24 h to adhere and were subsequently subjected to doxycycline treatments at 2 μg/ml. Doxycycline was replenished every 2 days to maintain continuity of the pTRIPZ system. Treatment was discontinued 7 days after induction. The cells were washed with ice-cold PBS and subsequently fixed with methanol. The cells were then stained with 0.5% crystal violet for 10 min, washed with water, and air-dried.

### Cell cycle analysis

The cells were subjected to 15 min of ethanol fixation at −20° C and subsequently collected by centrifugation. The cells were rehydrated at room temperature in PBS, pelleted, and resuspended in 3 μm 4′,6′-diamino-2-phenylindole diluted in staining buffer (100 mm Tris, pH 7.4, 150 mm NaCl, 1 mm CaCl_2_, 0.5 mm MgCl_2_, 0.1% Nonidet P-40). The cells were incubated for 15 min prior to flow cytometry by University of Michigan Flow Cytometry Core. Cell cycle distribution was evaluated using FCS Express.

### ChIP-sequencing library preparation and analysis

Native ChIP was conducted as previously described (47) using a mAb against CENPA (Abcam ab13939). CENPA-targeted ChIP DNA and input control DNA were prepared for parallel sequencing using the TruSeq ChIP library preparation kit (Illumina) according to the manufacturer's protocol. Library preparations were done in conjunction with the University of Michigan Sequencing Core. Paired-end libraries were sequenced with the Illumina HiSeq 4000 (2 × 150 nucleotide read length) with sequence coverage to >50 M total reads per sample. FastQC was employed to assess the overall quality of each sample followed by TrimGalore processing to trim low-quality bases and adapter sequences. The reads were aligned to hg38 using Bowtie2 (version 2.2.1) with default parameters. Principal-component analysis was conducted to determine the degree of variation between samples. PePr (version 1.1.14) was employed to identify CENPA bound regions. The *p* values were adjusted for multiple testing using the false discovery rate (FDR) approach, and CENPA-bound regions were considered significant peaks when *p* values < 1.0 × 10^−5^. The peaks were annotated with annotatr (version 1.0.3). Ontology assessments were conducted using ChIP-Enrich (48).

### RNA-sequencing library preparation and analysis

RNA was isolated as described and RNA integrity was evaluated using an Agilent TapeStation. Strand-specific libraries were prepared using the TruSeq stranded mRNA kit (Illumina) according to the manufacturer's protocol. Library preparations were done in conjunction with the University of Michigan Sequencing Core. Paired-end, strand-specific libraries were sequenced with the Illumina HiSeq 4000 (2 × 50 nucleotide read length) with sequence coverage to >50 M paired-end reads and >100 M total reads per sample. Sequencing results were run through a computational pipeline to trim low-quality bases and adapters (TrimGalore), align reads (STAR) to a reference genome (UCSC hg38 from iGenomes), quantify gene expression levels (HTseq), and call differentially expressed genes (edgeR). Ontology assessments were conducted using RNA-Enrich (49).

## Data availability

RNA-sequencing and ChIP-sequencing data are deposited in the NCBI's Gene Expression Omnibus with accession numbers GSE127835, GSE127836, and GSE127837. All other data are contained within the article.

## Supplementary Material

Supporting Information
