# Supplementary material for: The role of the histone H3 variant CENPA in prostate cancer
Source: J Biol Chem. 2020 May 5;295(25):8537–49. doi: 10.1074/jbc.RA119.010080 (PMC7307189; doi:10.1074/jbc.RA119.010080)
Supplement: Supporting Information [file supp_295_25_8537__index.html]

The role of the histone H3 variant CENPA in prostate cancer — Role of CENPA in prostate cancer — The role of the histone H3 variant CENPA in prostate cancer — Role of CENPA in prostate cancer — Supporting Information 

# The role of the histone H3 variant CENPA in prostate cancer

## Supporting Information

- Supporting Information (to be published online) - Supplemental Materials
- Supporting Information (to be published online) - Supplemental Dataset S1
- Supporting Information (to be published online) - Supplemental Dataset S2
- Supporting Information (to be published online) - Supplemental Dataset S3
- Supporting Information (to be published online) - Supplemental Dataset S4
- Supporting Information (to be published online) - Plasmid Specifications
