# Supplementary material for: The role of the histone H3 variant CENPA in prostate cancer
Source: J Biol Chem. 2020 May 5;295(25):8537–49. doi: 10.1074/jbc.RA119.010080 (PMC7307189; doi:10.1074/jbc.RA119.010080)
Supplement: Supporting Information [file supp_RA119.010080_154133_2_supp_523866_q9t4nb.pdf]

Non-canonical function of CENPA as a regulator of gene expression in prostate cancer

Anjan K. Saha, Rafael Contreras-Galindo, Yashar S. Niknafs, Matthew Iyer, Tingting Qin, Karthik Padmanabhan, Javed Siddiqui, Monica Palande, Claire Wang, Brian Qian, Elizabeth Ward, Tara Tang, Scott Tomlins, Scott Gitlin, Maureen Sartor, Gil S. Omenn, Arul M. Chinnaiyan, and David M. Markovitz

This PDF file includes:

Supplemental Figures 1 to 6
Captions for Supplemental Figures 1 to 6
Tables S1 to S3

Other supplementary materials for this manuscript include the following:

Datasets S1 to S4
Plasmid Specifications

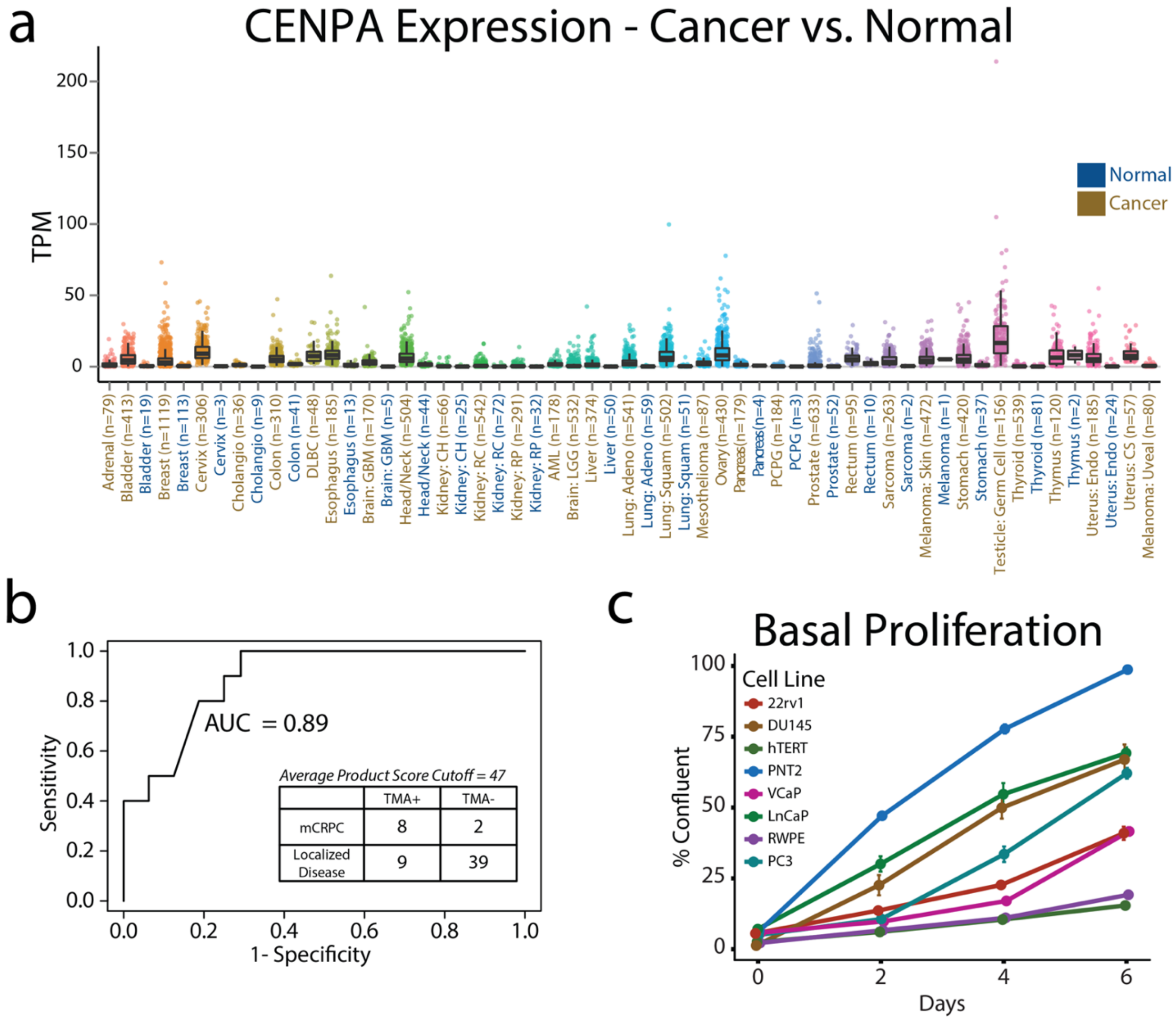

Supplementary Figure 1. Characterization of CENPA in cellular proliferation, and cancer. (A) Cancer vs. normal analysis conducted across a curated RNA-seq catalogue querying tissue CENPA levels by SSEA. (B) Receiver operator characteristic (ROC) separating metastatic castration resistant prostate cancer (mCRPC) from localized disease by CENPA staining in a tissue microarray. (C) Proliferation rates of a panel of prostate cancer (LnCaP, VCaP, 22rv1, DU145, and PC3) and benign prostatic epithelial (RWPE-1, 957E-hTERT, and PNT2) cell lines.

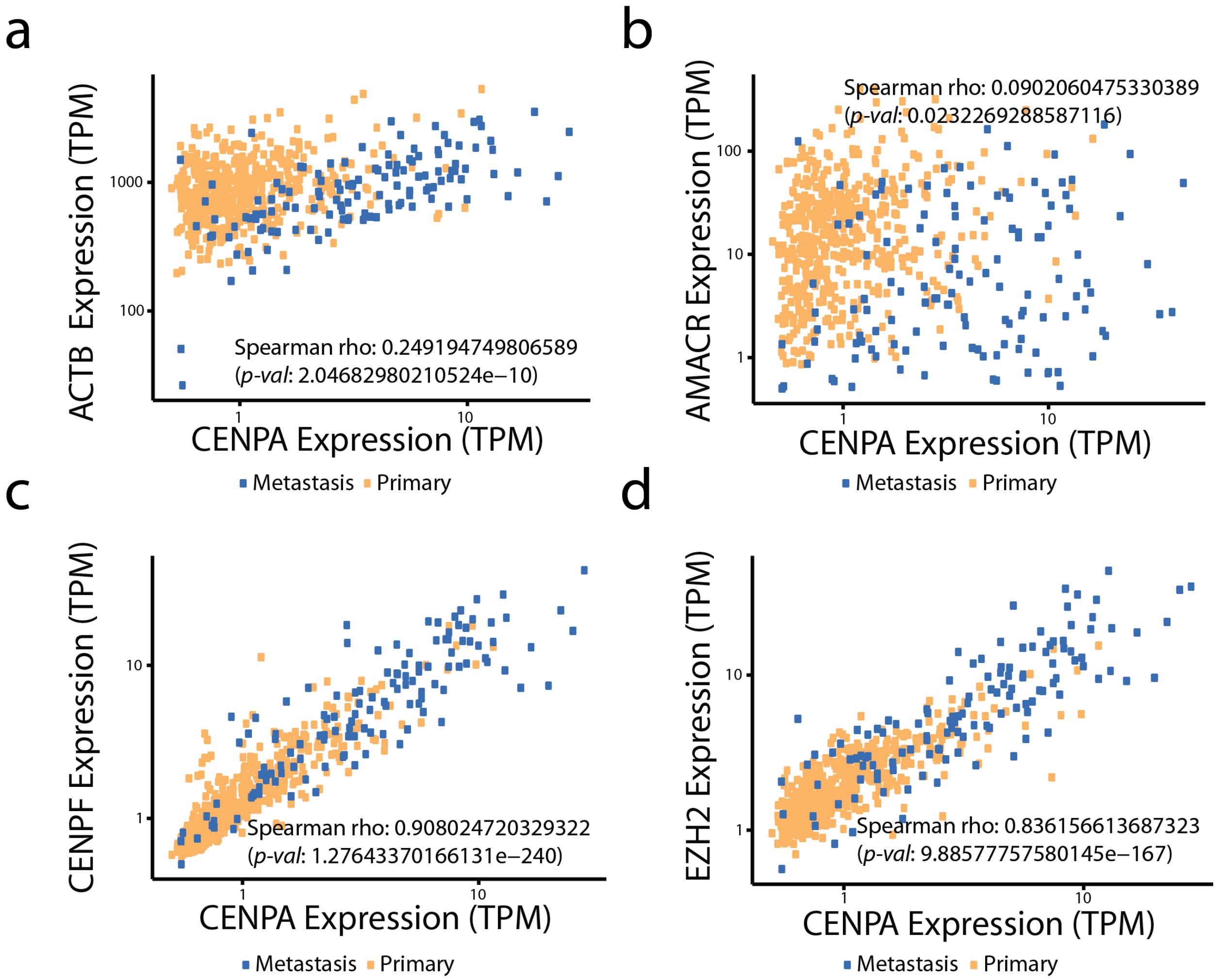

Supplementary Figure 2. Transcriptome-wide correlation against CENPA mRNA levels in prostate cancer. (A-D) Individual scatterplots depicting correlation strength between CENPA expression, previously characterized prostate cancer pathogenesis factors (AMACR, CENPF, and EZH2) and a housekeeping control gene (ACTB).

a

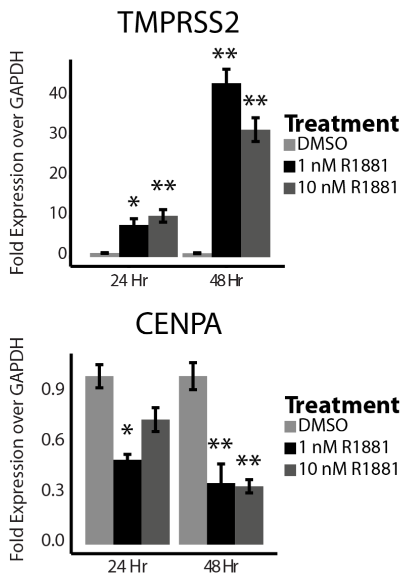

b

Label	Term	Count	p-value	FDR
GO: 0000777	Condensed Chromosome, Kinetochore	16	4.3×10^{-21}	5.1×10^{-20}
GO: 0000776	Kinetochore	16	4.6×10^{-19}	4.6×10^{-18}
UniProt Keyword	Kinetochore	18	1.7×10^{-24}	4.2×10^{-23}
GO: 0000779	Condensed Chromosome, Centromeric Region	18	8.8×10^{-24}	1.5×10^{-22}
GO: 0000793	Condensed Chromosome	23	2.3×10^{-26}	5.4×10^{-25}
GO: 0000775	Chromosome, Centromeric Region	23	8.9×10^{-27}	3.6×10^{-25}
GO: 0044427	Chromosomal Part	28	8.5×10^{-22}	1.1×10^{-20}
GO: 0005694	Chromosome	30	3.3×10^{-22}	5.0×10^{-21}

Annotation Cluster 2 *Enrichment Score (ES): 22.47*

c

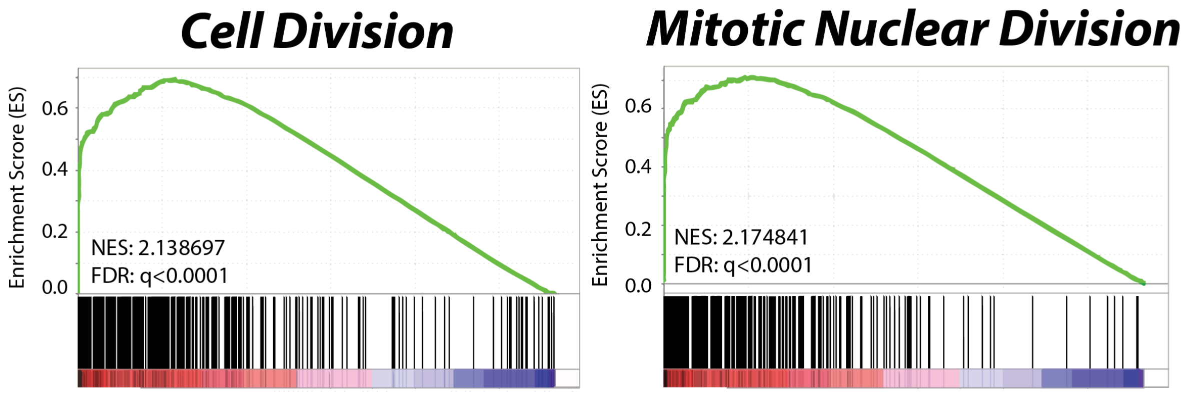

Supplementary Figure 3. Association between CENPA mRNA levels and cell division in prostate cancer. (A) VCaP prostate cancer cell lines treated with the androgen agonist R1881 and evaluated for CENPA and TMPRSS2 (androgen-responsive positive control) expression by qRT-PCR. *P<0.05, **P<0.01, comparing to DMSO for each condition and time point via Student's t-test. (B) Additional biological concepts identified as associated with CENPA expression through functional annotation analysis using DAVID, highlighting enrichments in concepts important for centromeric and kinetochore integrity. (C) Representative enrichment plots from GSEA of "Cell Division" (left) and "Mitotic Nuclear Division" (right) gene signatures conducted on transcriptome-wide correlation values pre-ranked by the strength of correlation.

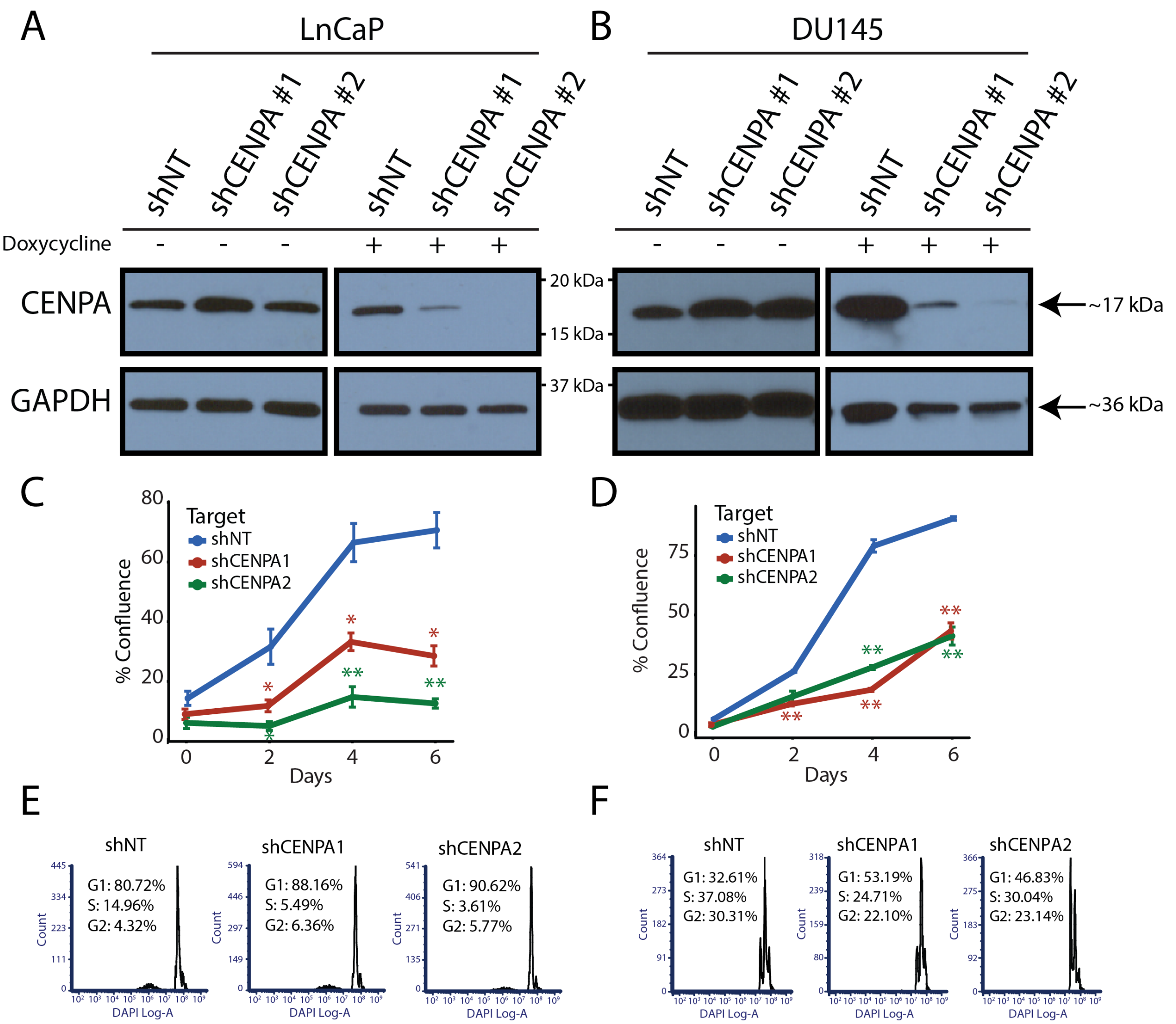

Supplementary Figure 4. Panel of prostate cancer cell lines subjected to CENPA depletion. (A, B) Immunoblot for CENPA and GAPDH in LnCaP and DU145 cells expressing a doxycycline inducible vector encoding a non-targeted and two independent CENPA-targeted shRNAs. (C, D) Growth curve depicting proliferation over 7 days following doxycycline induction in CENPA knockdown cell lines (left – LnCaP, right – DU145). Error bars represent the standard error of three biologic replicates. *P<0.05, **P<0.01, comparing to shNT for each condition via Student's t-test. (E, F) Cell cycle analysis with DAPI in CENPA shRNA-depleted LnCaP and DU145 cells compared to shNT.

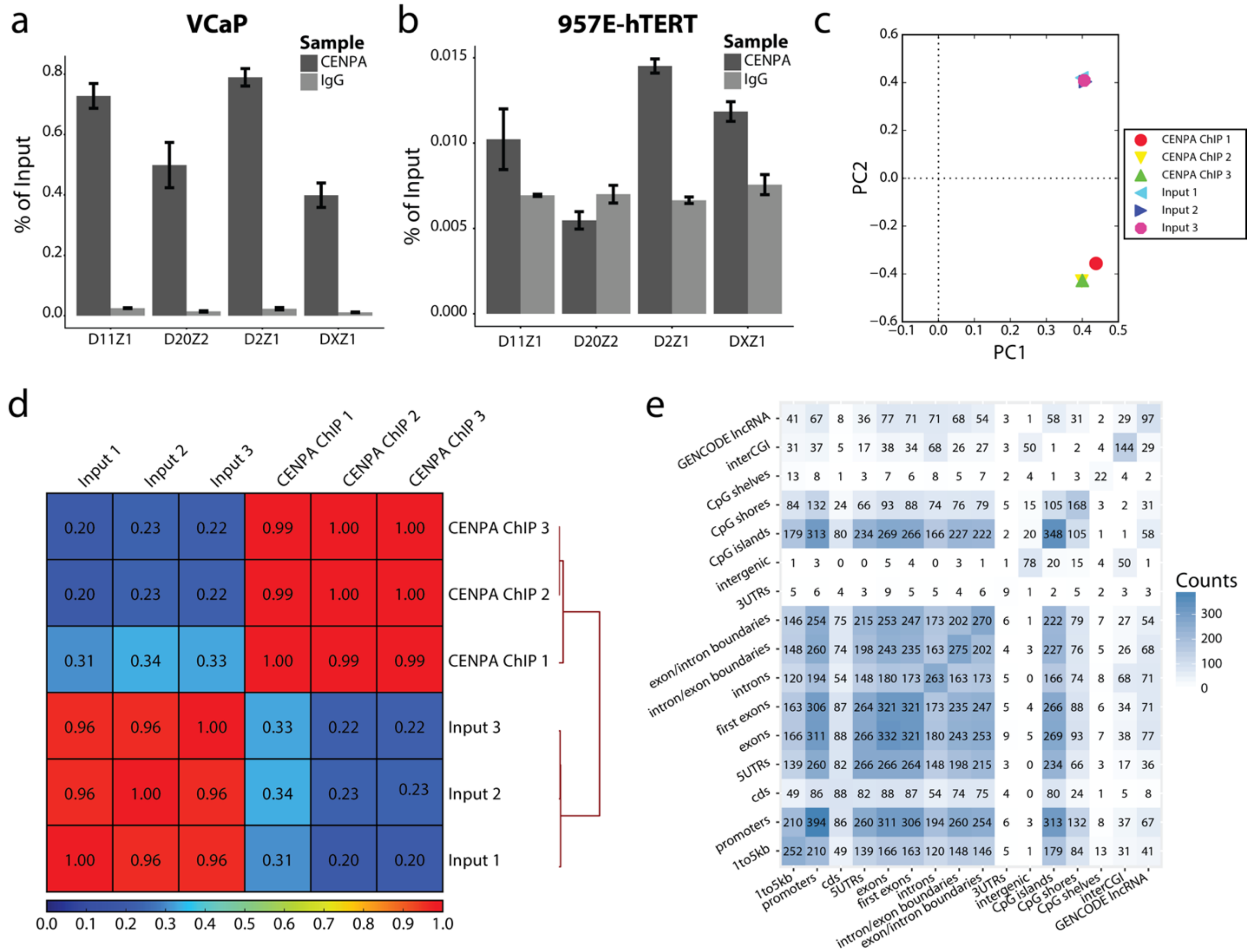

Supplementary Figure 5. CENPA N-ChIP-seq validation. (A) Validation of native chromatin immunoprecipitation (N-ChIP) efficiency of CENPA, through PCR targeting individual centromeric repeats. (B) N-ChIP-PCR conducted in benign prostatic epithelial cell line 957E-hTERT. (C) Principal Component Analysis (PCA) to determine variation in N-ChIP-seq replicates. (D) Heatmap depicting correlation strength between each individual N-ChIP-seq sample. Bottom left and top right indicate near perfect consistency between ChIP and input replicates, further validated by the unsupervised hierarchical clustering (see dendrogram along the vertical component). (E) Matrix depicting the degree of overlap of CENPA occupancy between different genomic regions. Abbreviations: CDS – coding sequence, UTR – untranslated region, CGI – CpG Island.

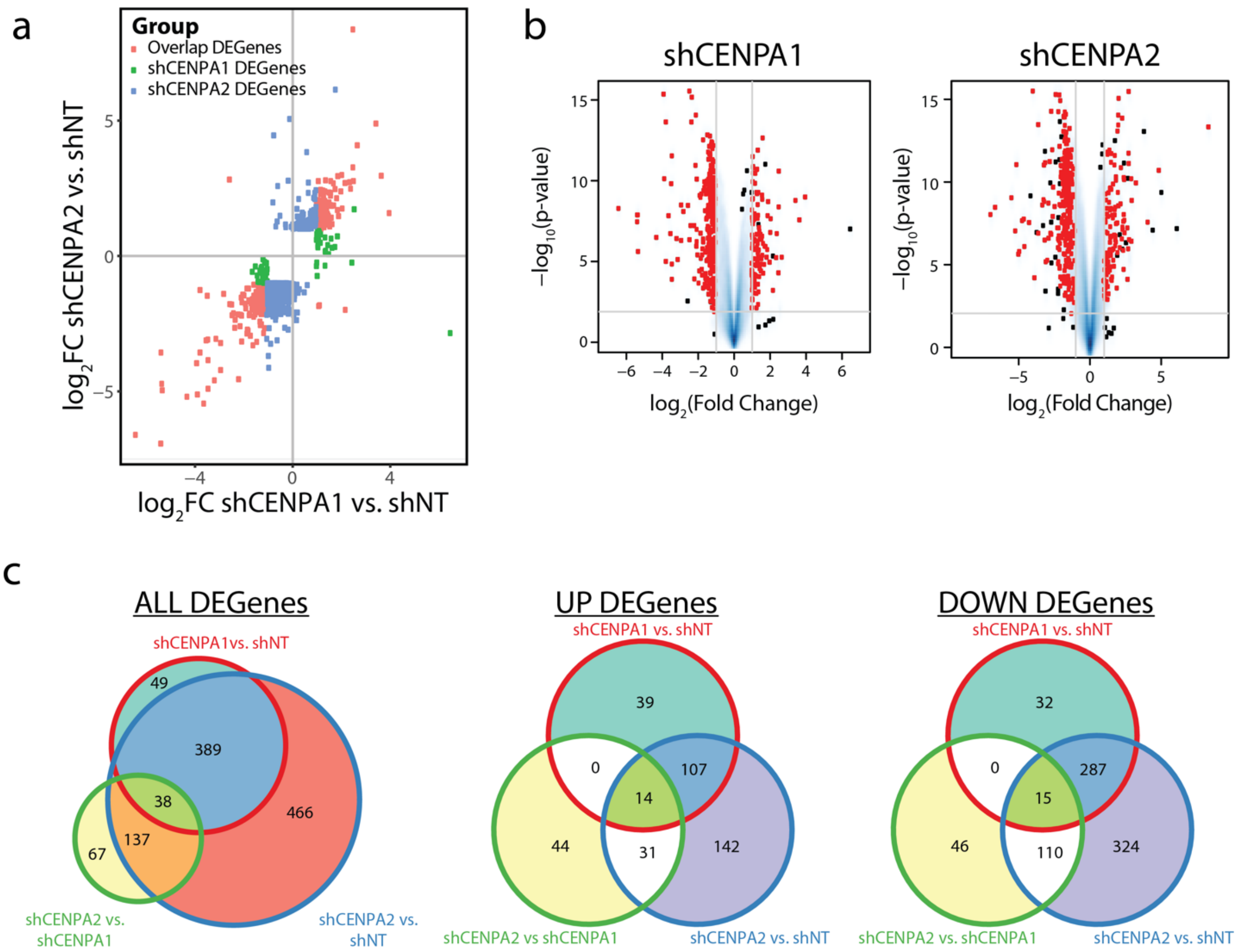

Supplementary Figure 6. CENPA-depletion RNA-seq quality control. (A) Scatterplot comparing the directionality of differentially expressed genes from the two independent CENPA-targeted shRNAs. (B) Volcano plot depicting significance against fold change (FC) between shNT and two independent CENPA-targeted shRNAs. Genes that satisfied the absolute FC > 1.5 (blue lines) and p-value < 0.01 (top right and top left) criteria were considered differentially expressed. (C) Venn diagrams illustrating the overlap in differential gene expression by comparison of analysis pairs for all differentially expressed genes (DEGs), upregulated DEGs and downregulated DEGs.

Table S1. Cancer vs. normal sample set enrichment analysis performed across tissue types for tissue CENPA expression

*CH: Chromophobe Renal Cell Carcinoma; GBM: Glioblastoma multiforme

Tissue Type	*Enrichment Score (ES)*	*Normalized ES (NES)*	*FDR*	*Percentile*
Uterus: Endometrial	0.824	4.45	<<<0.00001	0.993
Prostate	0.620	4.76	<<<0.00001	0.987
Kidney: CH*	0.544	2.61	0.00126	0.882
Colon	0.827	5.88	<<<0.00001	0.982
Bladder	0.754	3.98	<<<0.00001	0.995
Thyroid	0.277	2.61	0.000383	0.655
Lung: Squamous	0.986	7.85	<<<0.00001	0.999
Esophagus	0.865	3.59	<<<0.00001	0.997
Head/Neck	0.829	6.20	<<<0.00001	0.999
Stomach	0.770	5.35	<<<0.00001	0.992
Breast	0.796	9.42	<<<0.00001	0.996
Rectum	0.689	2.52	0.00277	0.906
Kidney: Renal Cell	0.758	6.88	<<<0.00001	0.975
Cholangiocarcinoma	1.00	3.38	<<<0.00001	0.867
Kidney: Renal Papillary	0.758	4.51	<<<0.00001	0.984
Lung: Adenocarcinoma	0.864	7.15	<<<0.00001	0.993
Liver	0.905	6.73	<<<0.00001	0.995
Brain: GBM*	1.00	2.66	3.06E-06	0.967

Table S2. Sample set enrichment analyses comparing expression levels of centromere and kinetochore genes in normal prostate tissues, primary prostate cancers, and metastatic prostate cancers.

Transcript ID	*Comparison Name*	*Enrichment Score (ES)*	*Normalized Enrichment Score (NES)*	*FDR*	*Percentile*
ENSG00000115163.13	Metastasis vs. Primary	0.677	7.64	<<<0.00001	0.982
ENSG00000115163.13	Metastasis vs. Normal	0.831	5.60	<<<0.00001	0.984
ENSG00000115163.13	Cancer vs. Normal	0.620	4.76	<<<0.00001	0.987
ENSG00000129810.13	Metastasis vs. Primary	0.734	8.11	<<<0.00001	0.988
ENSG00000129810.13	Metastasis vs. Normal	0.855	5.90	<<<0.00001	0.989
ENSG00000129810.13	Cancer vs. Normal	0.474	3.71	4.43E-07	0.946
ENSG00000117724.11	Metastasis vs. Primary	0.674	7.85	<<<0.00001	0.985
ENSG00000117724.11	Metastasis vs. Normal	0.832	5.96	<<<0.00001	0.990
ENSG00000117724.11	Cancer vs. Normal	0.552	4.37	<<<0.00001	0.976
ENSG00000123485.10	Metastasis vs. Primary	0.656	7.61	<<<0.00001	0.982
ENSG00000123485.10	Metastasis vs. Normal	0.845	5.73	<<<0.00001	0.986
ENSG00000123485.10	Cancer vs. Normal	0.643	5.05	<<<0.00001	0.992
ENSG00000138778.10	Metastasis vs. Primary	0.704	8.10	<<<0.00001	0.988
ENSG00000138778.10	Metastasis vs. Normal	0.875	6.13	<<<0.00001	0.993
ENSG00000138778.10	Cancer vs. Normal	0.450	3.64	4.43E-07	0.941
ENSG00000151725.10	Metastasis vs. Primary	0.605	7.06	<<<0.00001	0.975
ENSG00000151725.10	Metastasis vs. Normal	0.803	5.75	<<<0.00001	0.987
ENSG00000151725.10	Cancer vs. Normal	0.469	3.76	4.43E-07	0.948
ENSG00000102384.12	Metastasis vs. Primary	0.682	7.74	<<<0.00001	0.983
ENSG00000102384.12	Metastasis vs. Normal	0.849	5.84	<<<0.00001	0.988
ENSG00000102384.12	Cancer vs. Normal	0.309	2.42	0.00349	0.829

ENSG00000123219.11	Metastasis vs. Primary	0.655	7.49	<<<0.00001	0.981
ENSG00000123219.11	Metastasis vs. Normal	0.759	5.22	<<<0.00001	0.975
ENSG00000123219.11	Cancer vs. Normal	0.214	1.68	0.121	0.708

Table S3. Primers used in this study

Direction	***Target***	***Method***	***Sequence***
GAPDH F	GAPDH	qRT-PCR	ACATCGCTCAGACACCATG
GAPDH R	GAPDH	qRT-PCR	TGTAGTTGAGGTCAATGAAGGG
CENPA F	CENPA	qRT-PCR	GTGTGGACTTCAATTGGCAAG
CENPA R	CENPA	qRT-PCR	TGCACATCCTTTGGGAAGAG
TMPRSS2 F	TMPRSS2	qRT-PCR	CCTGCAGGGACATGGGCTATA
TMPRSS2 R	TMPRSS2	qRT-PCR	CCGGCACTTGTGTTCAGTTTC
D2Z1 F	D2Z1	ChIP-PCR	TCGTTGGAAACGGGATTGT
D2Z1 R	D2Z1	ChIP-PCR	CTGCTCTATGAAAGGGACTGTT
D11Z1 F	D11Z1	ChIP-PCR	CTTCCTTCGAAACGGGTATATCT
D11Z1 R	D11Z1	ChIP-PCR	GCTCCATCAGCAGGATTGT
DXZ1 F	DXZ1	ChIP-PCR	CGGGATCACCTTCCCATAAC
DXZ1 R	DXZ1	ChIP-PCR	GGTGTTGCAAACCTGAACTATC
D20Z2 F	D20Z2	ChIP-PCR	TGCTTGGAAACGGGAATGT
D20Z2 R	D20Z2	ChIP-PCR	CCTGCTCTATGAAAGGGAATGT
