# Supplementary material for: The role of the histone H3 variant CENPA in prostate cancer
Source: J Biol Chem. 2020 May 5;295(25):8537–49. doi: 10.1074/jbc.RA119.010080 (PMC7307189; doi:10.1074/jbc.RA119.010080)
Supplement: Supporting Information [file supp_RA119.010080_154133_2_supp_509551_q8l4fr.pdf]

Created with SnapGene®

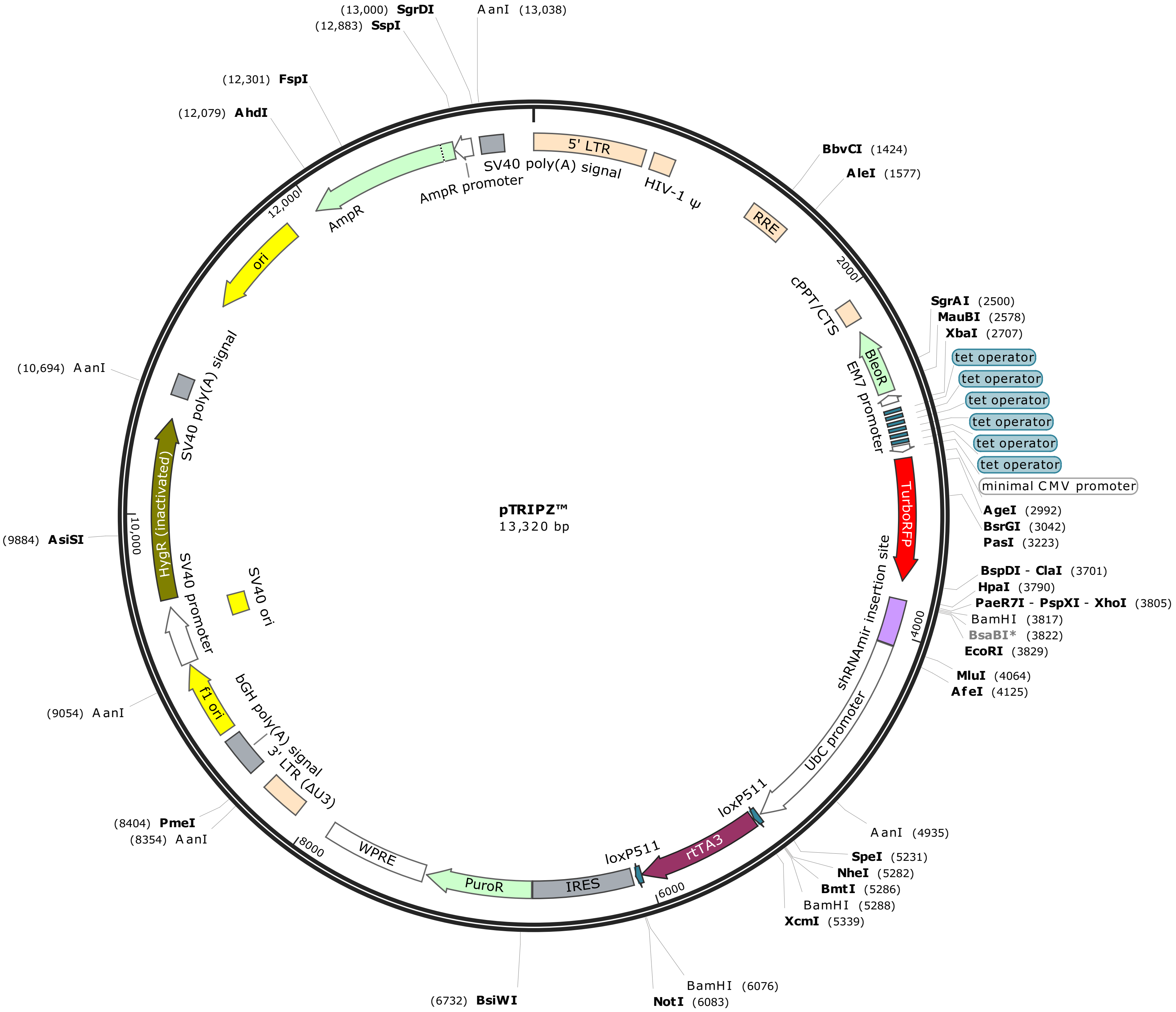

Vector Summary

**Vector ID**	VB170517-1083gzp
**Vector Name**	pLV[Exp]-mCherry-EF1A>{ORF_91bp}
**Date Created (Pacific Time)**	2017-05-17
**Size**	8796 bp
**Vector Type**	Lentivirus gene expression vector (3rd generation)
**Inserted Promoter**	EF1A
**Inserted ORF**	{ORF_91bp}
**Inserted Marker**	mCherry
**Plasmid Copy Number**	High
**Antibiotic Resistance**	Ampicillin
**Cloning Host**	Stbl3
**Vector Description**	*None*

Vector Map

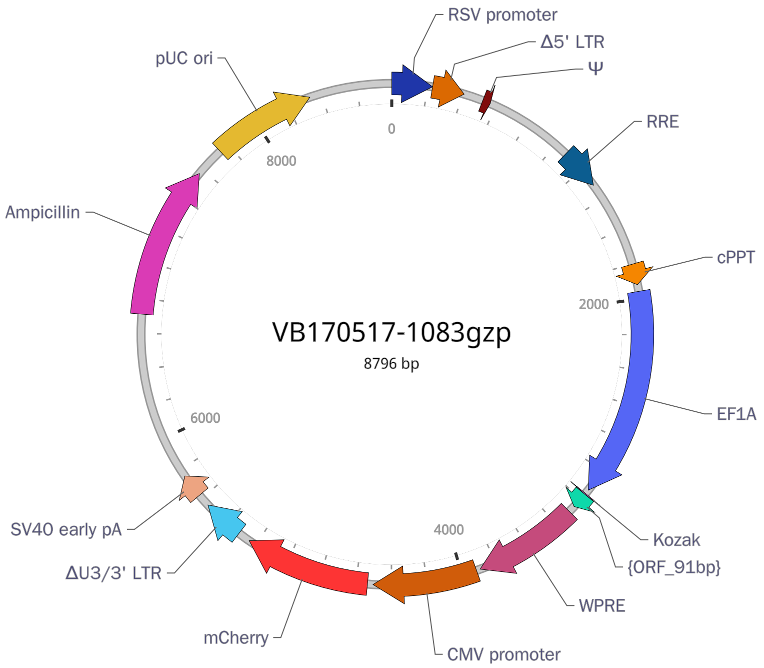

Vector Components

Name	Position	Size (bp)	Type	Description	Application Notes
RSV promoter	1-229	229	Promoter	Rous sarcoma virus enhancer/promoter	Strong promoter; drives transcription of viral RNA in packaging cells.
Δ5' LTR	230-410	181	LTR	Truncated HIV-1 5' long terminal repeat	Allows transcription of viral RNA and its packaging into virus.
Ψ	521-565	45	Miscellaneous	HIV-1 packaging signal	Allows packaging of viral RNA into virus.
RRE	1075-1308	234	Miscellaneous	HIV-1 Rev response element	Rev protein binding site that allows Rev-dependent nuclear export of viral RNA during viral packaging.
cPPT	1803-1920	118	Miscellaneous	Central polypurine tract	Facilitates the nuclear import of HIV-1 cDNA through a central DNA flap.
**EF1A**	1959-3137	1179	Promoter	Human eukaryotic translation elongation factor 1 α1 promoter	Strong promoter.
Kozak	3162-3167	6	Miscellaneous	Kozak translation initiation sequence	Facilitates translation initiation of ATG start codon downstream of the Kozak sequence.
**{ORF_91bp}**	3168-3258	91	ORF	*None*	*None*
WPRE	3297-3894	598	Miscellaneous	Woodchuck hepatitis virus posttranscriptional regulatory element	Enhances viral RNA stability in packaging cells, leading to higher titer of packaged virus.
CMV promoter	3916-4503	588	Promoter	Human cytomegalovirus immediate early enhancer/promoter	Strong promoter; may have variable strength in some cell types.
**mCherry**	4535-5245	711	ORF	Variant of mRFP1 generated by mutagenesis	Commonly used red fluorescent protein; fast maturation compared to its predecessor, mRFP1.
ΔU3/3' LTR	5316-5550	235	LTR	Truncated HIV-1 3' long terminal repeat	Allows packaging of viral RNA into virus; self-inactivates the 5' LTR by a copying mechanism during viral genome integration; contains polyadenylation signal for transcription termination.
SV40 early pA	5623-5757	135	PolyA_signal	Simian virus 40 early polyadenylation signal	Allows transcription termination and polyadenylation of mRNA transcribed by Pol II RNA polymerase.
Ampicillin	6711-7571	861	ORF	Ampicillin resistance gene	Allows E. coli to be resistant to ampicillin.
pUC ori	7742-8330	589	rep_origin	pUC origin of replication	Facilitates plasmid replication in E. coli; regulates high-copy plasmid number (500-700).

Note: Components added by user are listed in **bold red** text.

Vector Sequence

```
  1 AATGTAGTCT TATGCAATAC TCTTGTAGTC TTGCAACATG GTAACGATGA GTTAGCAACA TGCCTTACAA GGAGAGAAAA AGCACCGTGC ATGCCGATTG
101 GTGGAAGTAA GGTGGTACGA TCGTGCCTTA TTAGGAAGGC AACAGACGGG TCTGACATGG ATTGGACGAA CCACTGAATT GCCGCATTGC AGAGATATTG
201 TATTTAAGTG CCTAGCTCGA TACATAAACG GGTCTCTCTG GTTAGACCAG ATCTGAGCCT GGGAGCTCTC TGGCTAACTA GGGAACCCAC TGCTTAAGCC
301 TCAATAAAGC TTGCCTTGAG TGCTTCAAGT AGTGTGTGCC CGTCTGTTGT GTGACTCTGG TAACTAGAGA TCCCTCAGAC CCTTTTAGTC AGTGTGGAAA
401 ATCTCTAGCA GTGGCGCCCG AACAGGGACT TGAAAGCGAA AGGGAAACCA GAGGAGCTCT CTCGACGCAG GACTCGGCTT GCTGAAGCGC GCACGGCAAG
501 AGGCGAGGGG CGGCGACTGG TGAGTACGCC AAAAATTTTG ACTAGCGGAG GCTAGAAGGA GAGAGATGGG TGCGAGAGCG TCAGTATTAA GCGGGGGAGA
601 ATTAGATCGC GATGGGAAAA AATTCGGTTA AGGCCAGGGG GAAAGAAAAA ATATAAATTA AAACATATAG TATGGGCAAG CAGGGAGCTA GAACGATTCG
701 CAGTTAATCC TGGCCTGTTA GAAACATCAG AAGGCTGTAG ACAAATACTG GGACAGCTAC AACCATCCCT TCAGACAGGA TCAGAAGAAC TTAGATCATT
```

```
801  ATATAATACA GTAGCAACCC TCTATTGTGT GCATCAAAGG ATAGAGATAA AAGACACCAA GGAAGCTTTA GACAAGATAG AGGAAGAGCA AAACAAAAGT
901  AAGACCACCG CACAGCAAGC GGCCGCTGAT CTTCAGACCT GGAGGAGGAG ATATGAGGGA CAATTGGAGA AGTGAATTAT ATAAATATAA AGTAGTAAAA
1001 ATTGAACCAT TAGGAGTAGC ACCCACCAAG GCAAAGAGAA GAGTGGTGCA GAGAGAAAAA AGAGCAGTGG GAATAGGAGC TTTGTTCCTT GGGTTCTTGG
1101 GAGCAGCAGG AAGCACTATG GGCGCAGCGT CAATGACGCT GACGGTACAG GCCAGACAAT TATTGTCTGG TATAGTGCAG CAGCAGAACA ATTTGCTGAG
1201 GGCTATTGAG GCGCAACAGC ATCTGTTGCA ACTCACAGTC TGGGGCATCA AGCAGCTCCA GGCAAGAATC CTGGCTGTGG AAAGATACCT AAAGGATCAA
1301 CAGCTCCTGG GGATTTGGGG TTGCTCTGGA AAACTCATTT GCACCACTGC TGTGCCTTGG AATGCTAGTT GGAGTAATAA ATCTCTGGAA CAGATTTGGA
1401 ATCACACGAC CTGGATGGAG TGGGACAGAG AAATTAACAA TTACACAAGC TTAATACACT CCTTAATTGA AGAATCGCAA AACCAGCAAG AAAAGAATGA
1501 ACAAGAATTA TTGGAATTAG ATAAATGGGC AAGTTTGTGG AATTGGTTTA ACATAACAAA TTGGCTGTGG TATATAAAAT TATTCATAAT GATAGTAGGA
1601 GGCTTGGTAG GTTTAAGAAT AGTTTTTGCT GTACTTTCTA TAGTGAATAG AGTTAGGCAG GGATATTCAC CATTATCGTT TCAGACCCAC CTCCCAACCC
1701 CGAGGGGACC CGACAGGCCC GAAGGAATAG AAGAAGAAGG TGGAGAGAGA GACAGAGACA GATCCATTCG ATTAGTGAAC GGATCTCGAC GGTATCGCTA
1801 GCTTTTAAAA GAAAAGGGGG GATTGGGGGG TACAGTGCAG GGGAAAGAAT AGTAGACATA ATAGCAACAG ACATACAAAC TAAAGAATTA CAAAAACAAA
1901 TTACAAAAAT TCAAAATTTT ACTAGTGATT ATCGGATCAA CTTTGTATAG AAAAGTTGGG CTCCGGTGCC CGTCAGTGGG CAGAGCGCAC ATCGCCCACA
2001 GTCCCCGAGA AGTTGGGGGG AGGGGTCGGC AATTGAACCG GTGCCTAGAG AAGGTGGCGC GGGGTAAACT GGGAAAGTGA TGTCGTGTAC TGGCTCCGCC
2101 TTTTTCCCGA GGGTGGGGGA GAACCGTATA TAAGTGCAGT AGTCGCCGTG AACGTTCTTT TTCGCAACGG GTTTGCCGCC AGAACACAGG TAAGTGCCGT
2201 GTGTGGTTCC CGCGGGCCTG GCCTCTTTAC GGGTTATGGC CCTTGCGTGC CTTGAATTAC TTCCACCTGG CTGCAGTACG TGATTCTTGA TCCCGAGCTT
2301 CGGGTTGGAA GTGGGTGGGA GAGTTCGAGG CCTTGCGCTT AAGGAGCCCC TTCGCCTCGT GCTTGAGTTG AGGCCTGGCC TGGGCGCTGG GGCCGCCGCG
2401 TGCGAATCTG GTGGCACCTT CGCGCCTGTC TCGCTGCTTT CGATAAGTCT CTAGCCATTT AAAATTTTTG ATGACCTGCT GCGACGCTTT TTTTCTGGCA
2501 AGATAGTCTT GTAAATGCGG GCCAAGATCT GCACACTGGT ATTTCGGTTT TTGGGGCCGC GGGCGGCGAC GGGGCCCGTG CGTCCCAGCG CACATGTTCG
2601 GCGAGGCGGG GCCTGCGAGC GCGGCCACCG AGAATCGGAC GGGGGTAGTC TCAAGCTGGC CGGCCTGCTC TGGTGCCTGG TCTCGCGCCG CCGTGTATCG
2701 CCCCGCCCTG GGCGGCAAGG CTGGCCCGGT CGGCACCAGT TGCGTGAGCG GAAAGATGGC CGCTTCCCGG CCCTGCTGCA GGGAGCTCAA AATGGAGGAC
2801 GCGGCGCTCG GGAGAGCGGG CGGGTGAGTC ACCCACACAA AGGAAAAGGG CCTTTCCGTC CTCAGCCGTC GCTTCATGTG ACTCCACGGA GTACCGGGCG
2901 CCGTCCAGGC ACCTCGATTA GTTCTCGAGC TTTTGGAGTA CGTCGTCTTT AGGTTGGGGG GAGGGGTTTT ATGCGATGGA GTTTCCCCAC ACTGAGTGGG
3001 TGGAGACTGA AGTTAGGCCA GCTTGGCACT TGATGTAATT CTCCTTGGAA TTTGCCCTTT TTGAGTTTGG ATCTTGGTTC ATTCTCAAGC CTCAGACAGT
3101 GGTTCAAAGT TTTTTTCTTC CATTTCAGGT GTCGTGACAA GTTTGTACAA AAAAGCAGGC TGCCACCAAT TCGAATTCCT AGCGGATCCA ATTGCTCGAG
3201 CGAAAGCGGC CGCGAAAGTC GACAATTAAT TAACGTTAAC AAACCGGTAA TCGATAACAC CCAGCTTTCT TGTACAAAGT GGTGATAATC GAATTCCGAT
3301 AATCAACCTC TGGATTACAA AATTTGTGAA AGATTGACTG GTATTCTTAA CTATGTTGCT CCTTTTACGC TATGTGGATA CGCTGCTTTA ATGCCTTTGT
3401 ATCATGCTAT TGCTTCCCGT ATGGCTTTCA TTTTCTCCTC CTTGTATAAA TCCTGGTTGC TGTCTCTTTA TGAGGAGTTG TGGCCCGTTG TCAGGCAACG
3501 TGGCGTGGTG TGCACTGTGT TTGCTGACGC AACCCCCACT GGTTGGGGCA TTGCCACCAC CTGTCAGCTC CTTTCCGGGA CTTTCGCTTT CCCCCTCCCT
3601 ATTGCCACGG CGGAACTCAT CGCCGCCTGC CTTGCCCGCT GCTGGACAGG GGCTCGGCTG TTGGGCACTG ACAATTCCGT GGTGTTGTCG GGGAAGCTGA
3701 CGTCCTTTCC ATGGCTGCTC GCCTGTGTTG CCACCTGGAT TCTGCGCGGG ACGTCCTTCT GCTACGTCCC TTCGGCCCTC AATCCAGCGG ACCTTCCTTC
3801 CCGCGGCCTG CTGCCGGCTC TGCGGCCTCT TCCGCGTCTT CGCCTTCGCC CTCAGACGAG TCGGATCTCC CTTTGGGCCG CCTCCCCGCA TCGGGAATTC
3901 CCGCGGTTCG AACGCGTTGA CATTGATTAT TGACTAGTTA TTAATAGTAA TCAATTACGG GGTCATTAGT TCATAGCCCA TATATGGAGT TCCGCGTTAC
4001 ATAACTTACG GTAAATGGCC CGCCTGGCTG ACCGCCCAAC GACCCCCGCC CATTGACGTC AATAATGACG TATGTTCCCA TAGTAACGCC AATAGGGACT
4101 TTCCATTGAC GTCAATGGGT GGAGTATTTA CGGTAAACTG CCCACTTGGC AGTACATCAA GTGTATCATA TGCCAAGTAC GCCCCCTATT GACGTCAATG
4201 ACGGTAAATG GCCCGCCTGG CATTATGCCC AGTACATGAC CTTATGGGAC TTTCCTACTT GGCAGTACAT CTACGTATTA GTCATCGCTA TTACCATGGT
4301 GATGCGGTTT TGGCAGTACA TCAATGGGCG TGGATAGCGG TTTGACTCAC GGGGATTTCC AAGTCTCCAC CCCATTGACG TCAATGGGAG TTTGTTTTGG
4401 CACCAAAATC AACGGGACTT TCCAAAATGT CGTAACAACT CCGCCCCATT GACGCAAATG GGCGGTAGGC GTGTACGGTG GGAGGTCTAT ATAAGCAGAG
4501 CTCTCTGGCT AACTAGAGAA CCCACTGCGC CACCATGGTG AGCAAGGGCG AGGAGGATAA CATGGCCATC ATCAAGGAGT TCATGCGCTT CAAGGTGCAC
4601 ATGGAGGGCT CCGTGAACGG CCACGAGTTC GAGATCGAGG GCGAGGGCGA GGGCCGCCCC TACGAGGGCA CCCAGACCGC CAAGCTGAAG GTGACCAAGG
4701 GTGGCCCCCT GCCCTTCGCC TGGGACATCC TGTCCCCTCA GTTCATGTAC GGCTCCAAGG CCTACGTGAA GCACCCCGCC GACATCCCCG ACTACTTGAA
4801 GCTGTCCTTC CCCGAGGGCT TCAAGTGGGA GCGCGTGATG AACTTCGAGG ACGGCGGCGT GGTGACCGTG ACCCAGGACT CCTCCCTGCA GGACGGCGAG
4901 TTCATCTACA AGGTGAAGCT GCGCGGCACC AACTTCCCCT CCGACGGCCC CGTAATGCAG AAGAAGACCA TGGGCTGGGA GGCCTCCTCC GAGCGGATGT
5001 ACCCCGAGGA CGGCGCCCTG AAGGGCGAGA TCAAGCAGAG GCTGAAGCTG AAGGACGGCG GCCACTACGA CGCTGAGGTC AAGACCACCT ACAAGGCCAA
5101 GAAGCCCGTG CAGCTGCCCG GCGCCTACAA CGTCAACATC AAGTTGGACA TCACCTCCCA CAACGAGGAC TACACCATCG TGGAACAGTA CGAACGCGCC
5201 GAGGGCCGCC ACTCCACCGG CGGCATGGAC GAGCTGTACA AGTAAGGTAC CTTTAAGACC AATGACTTAC AAGGCAGCTG TAGATCTTAG CCACTTTTTA
5301 AAAGAAAAGG GGGGACTGGA AGGGCTAATT CACTCCCAAC GAAGACAAGA TCTGCTTTTT GCTTGTACTG GGTCTCTCTG GTTAGACCAG ATCTGAGCCT
5401 GGGAGCTCTC TGGCTAACTA GGGAACCCAC TGCTTAAGCC TCAATAAAGC TTGCCTTGAG TGCTTCAAGT AGTGTGTGCC CGTCTGTTGT GTGACTCTGG
5501 TAACTAGAGA TCCCTCAGAC CCTTTTAGTC AGTGTGGAAA ATCTCTAGCA GTAGTAGTTC ATGTCATCTT ATTATTCAGT ATTTATAACT TGCAAAGAAA
5601 TGAATATCAG AGAGTGAGAG GAACTTGTTT ATTGCAGCTT ATAATGGTTA CAAATAAAGC AATAGCATCA CAAATTTCAC AAATAAAGCA TTTTTTTCAC
5701 TGCATTCTAG TTGTGGTTTG TCCAAACTCA TCAATGTATC TTATCATGTC TGGCTCTAGC TATCCCGCCC CTAACTCCGC CCATCCCGCC CCTAACTCCG
5801 CCCAGTTCCG CCCATTCTCC GCCCCATGGC TGACTAATTT TTTTTATTTA TGCAGAGGCC GAGGCCGCCT CGGCCTCTGA GCTATTCCAG AAGTAGTGAG
5901 GAGGCTTTTT TGGAGGCCTA GGGACGTACC CAATTCGCCC TATAGTGAGT CGTATTACGC GCGCTCACTG GCCGTCGTTT TACAACGTCG TGACTGGGAA
6001 AACCCTGGCG TTACCCAACT TAATCGCCTT GCAGCACATC CCCCTTTCGC CAGCTGGCGT AATAGCGAAG AGGCCCGCAC CGATCGCCCT TCCCAACAGT
6101 TGCGCAGCCT GAATGGCGAA TGGGACGCGC CCTGTAGCGG CGCATTAAGC GCGGCGGGTG TGGTGGTTAC GCGCAGCGTG ACCGCTACAC TTGCCAGCGC
6201 CCTAGCGCCC GCTCCTTTCG CTTTCTTCCC TTCCTTTCTC GCCACGTTCG CCGGCTTTCC CCGTCAAGCT CTAAATCGGG GGCTCCCTTT AGGGTTCCGA
6301 TTTAGTGCTT TACGGCACCT CGACCCCAAA AAACTTGATT AGGGTGATGG TTCACGTAGT GGGCCATCGC CCTGATAGAC GGTTTTTCGC CCTTTGACGT
6401 TGGAGTCCAC GTTCTTTAAT AGTGGACTCT TGTTCCAAAC TGGAACAACA CTCAACCCTA TCTCGGTCTA TTCTTTTGAT TTATAAGGGA TTTTGCCGAT
6501 TTCGGCCTAT TGGTTAAAAA ATGAGCTGAT TTAACAAAAA TTTAACGCGA ATTTTAACAA AATATTAACG CTTACAATTT AGGTGGCACT TTTCGGGGAA
6601 ATGTGCGCGG AACCCCTATT TGTTTATTTT TCTAAATACA TTCAAATATG TATCCGCTCA TGAGACAATA ACCCTGATAA ATGCTTCAAT AATATTGAAA
6701 AAGGAAGAGT ATGAGTATTC AACATTTCCG TGTCGCCCTT ATTCCCTTTT TTGCGGCATT TTGCCTTCCT GTTTTTGCTC ACCCAGAAAC GCTGGTGAAA
6801 GTAAAAGATG CTGAAGATCA GTTGGGTGCA CGAGTGGGTT ACATCGAACT GGATCTCAAC AGCGGTAAGA TCCTTGAGAG TTTTCGCCCC GAAGAACGTT
6901 TTCCAATGAT GAGCACTTTT AAAGTTCTGC TATGTGGCGC GGTATTATCC CGTATTGACG CCGGGCAAGA GCAACTCGGT CGCCGCATAC ACTATTCTCA
7001 GAATGACTTG GTTGAGTACT CACCAGTCAC AGAAAAGCAT CTTACGGATG GCATGACAGT AAGAGAATTA TGCAGTGCTG CCATAACCAT GAGTGATAAC
7101 ACTGCGGCCA ACTTACTTCT GACAACGATC GGAGGACCGA AGGAGCTAAC CGCTTTTTTG CACAACATGG GGGATCATGT AACTCGCCTT GATCGTTGGG
7201 AACCGGAGCT GAATGAAGCC ATACCAAACG ACGAGCGTGA CACCACGATG CCTGTAGCAA TGGCAACAAC GTTGCGCAAA CTATTAACTG GCGAACTACT
7301 TACTCTAGCT TCCCGGCAAC AATTAATAGA CTGGATGGAG GCGGATAAAG TTGCAGGACC ACTTCTGCGC TCGGCCCTTC CGGCTGGCTG GTTTATTGCT
7401 GATAAATCTG GAGCCGGTGA GCGTGGGTCT CGCGGTATCA TTGCAGCACT GGGGCCAGAT GGTAAGCCCT CCCGTATCGT AGTTATCTAC ACGACGGGGA
7501 GTCAGGCAAC TATGGATGAA CGAAATAGAC AGATCGCTGA GATAGGTGCC TCACTGATTA AGCATTGGTA ACTGTCAGAC CAAGTTTACT CATATATACT
7601 TTAGATTGAT TTAAAACTTC ATTTTTAATT TAAAAGGATC TAGGTGAAGA TCCTTTTTGA TAATCTCATG ACCAAAATCC CTTAACGTGA GTTTTCGTTC
7701 CACTGAGCGT CAGACCCCGT AGAAAAGATC AAAGGATCTT CTTGAGATCC TTTTTTTCTG CGCGTAATCT GCTGCTTGCA AACAAAAAAA CCACCGCTAC
7801 CAGCGGTGGT TTGTTTGCCG GATCAAGAGC TACCAACTCT TTTTCCGAAG GTAACTGGCT TCAGCAGAGC GCAGATACCA AATACTGTTC TTCTAGTGTA
7901 GCCGTAGTTA GGCCACCACT TCAAGAACTC TGTAGCACCG CCTACATACC TCGCTCTGCT AATCCTGTTA CCAGTGGCTG CTGCCAGTGG CGATAAGTCG
8001 TGTCTTACCG GGTTGGACTC AAGACGATAG TTACCGGATA AGGCGCAGCG GTCGGGCTGA ACGGGGGGTT CGTGCACACA GCCCAGCTTG GAGCGAACGA
8101 CCTACACCGA ACTGAGATAC CTACAGCGTG AGCTATGAGA AAGCGCCACG CTTCCCGAAG AGAGAAAGGC GGACAGGTAT CCGGTAAGCG GCAGGGTCGG
8201 AACAGGAGAG CGCACGAGGG AGCTTCCAGG GGGAAACGCC TGGTATCTTT ATAGTCCTGT CGGGTTTCGC CACCTCTGAC TTGAGCGTCG ATTTTTGTGA
8301 TGCTCGTCAG GGGGGCGGAG CCTATGGAAA AACGCCAGCA ACGCGGCCTT TTTACGGTTC CTGGCCTTTT GCTGGCCTTT TGCTCACATG TTCTTTCCTG
8401 CGTTATCCCC TGATTCTGTG GATAACCGTA TTACCGCCTT TGAGTGAGCT GATACCGCTC GCCGCAGCCG AACGACCGAG CGCAGCGAGT CAGTGAGCGA
8501 GGAAGCGGAA GAGCGCCCAA TACGCAAACC GCCTCTCCCC GCGCGTTGGC CGATTCATTA ATGCAGCTGG CACGACAGGT TTCCCGACTG GAAAGCGGGC
8601 AGTGAGCGCA ACGCAATTAA TGTGAGTTAG CTCACTCATT AGGCACCCCA GGCTTTACAC TTTATGCTTC CGGCTCGTAT GTTGTGTGGA ATTGTGAGCG
8701 GATAACAATT TCACACAGGA AACAGCTATG ACCATGATTA CGCCAAGCGC GCAATTAACC CTCACTAAAG GGAACAAAAG CTGGAGCTGC AAGCTT
```

Validation by Restriction Enzyme Digestion

Cutters	Locations	Fragments (bp)
**PacI**	3231	8796
**HpaI**	3238	8796
**ApaLI**	3511, 4596, 6827, 8073	1085, 2231, 1246, 4234
**BamHI**	3185	8796
**EcoRI**	3174, 3292, 3896	118, 604, 8074
**ApaLI+HpaI**	3238, 3511, 4596, 6827, 8073	273, 1085, 2231, 1246, 3961
**ApaLI+PacI**	3231, 3511, 4596, 6827, 8073	280, 1085, 2231, 1246, 3954
**ApaLI+BamHI**	3185, 3511, 4596, 6827, 8073	326, 1085, 2231, 1246, 3908
**ApaLI+EcoRI**	3174, 3292, 3511, 3896, 4596, 6827, 8073	118, 219, 385, 700, 2231, 1246, 3897

Vector Summary

Vector ID	VB170517-1081qhz
Vector Name	pLV[Exp]-mCherry-EF1A>hCENPA[NM_001809.3]
Date Created (Pacific Time)	2017-05-17
Size	9128 bp
Vector Type	Lentivirus gene expression vector (3rd generation)
Inserted Promoter	EF1A
Inserted ORF	hCENPA[NM_001809.3]
Inserted Marker	mCherry
Plasmid Copy Number	High
Antibiotic Resistance	Ampicillin
Cloning Host	Stbl3
Vector Description	*None*

Vector Map

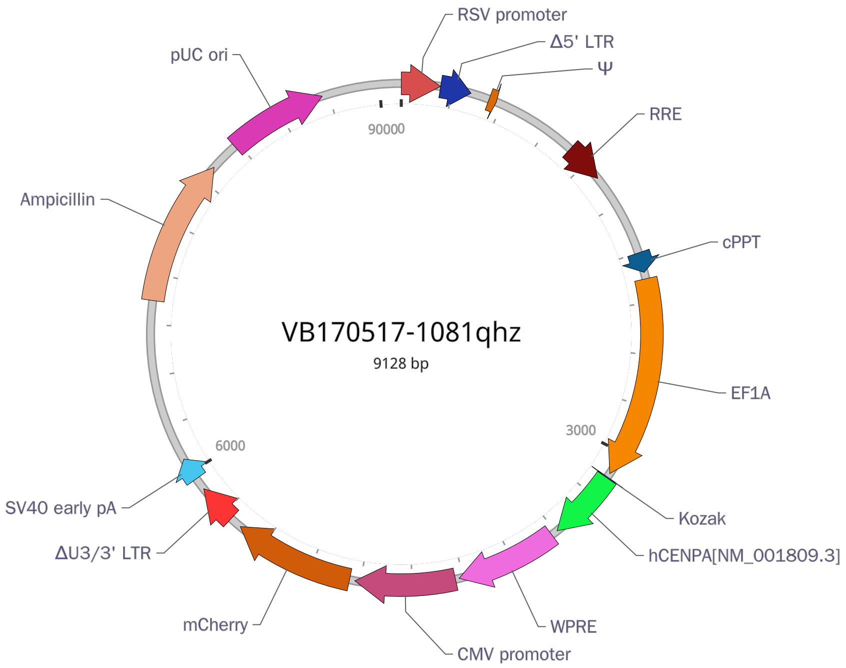

Vector Components

Name	Position	Size (bp)	Type	Description	Application Notes
RSV promoter	1-229	229	Promoter	Rous sarcoma virus enhancer/promoter	Strong promoter; drives transcription of viral RNA in packaging cells.
Δ5' LTR	230-410	181	LTR	Truncated HIV-1 5' long terminal repeat	Allows transcription of viral RNA and its packaging into virus.
Ψ	521-565	45	Miscellaneous	HIV-1 packaging signal	Allows packaging of viral RNA into virus.
RRE	1075-1308	234	Miscellaneous	HIV-1 Rev response element	Rev protein binding site that allows Rev-dependent nuclear export of viral RNA during viral packaging.
cPPT	1803-1920	118	Miscellaneous	Central polypurine tract	Facilitates the nuclear import of HIV-1 cDNA through a central DNA flap.
**EF1A**	1959-3137	1179	Promoter	Human eukaryotic translation elongation factor 1 α1 promoter	Strong promoter.
Kozak	3162-3167	6	Miscellaneous	Kozak translation initiation sequence	Facilitates translation initiation of ATG start codon downstream of the Kozak sequence.
**hCENPA[NM_001809.3]**	3168-3590	423	ORF	*None*	*None*
WPRE	3629-4226	598	Miscellaneous	Woodchuck hepatitis virus posttranscriptional regulatory element	Enhances viral RNA stability in packaging cells, leading to higher titer of packaged virus.
CMV promoter	4248-4835	588	Promoter	Human cytomegalovirus immediate early enhancer/promoter	Strong promoter; may have variable strength in some cell types.
**mCherry**	4867-5577	711	ORF	Variant of mRFP1 generated by mutagenesis	Commonly used red fluorescent protein; fast maturation compared to its predecessor, mRFP1.
ΔU3/3' LTR	5648-5882	235	LTR	Truncated HIV-1 3' long terminal repeat	Allows packaging of viral RNA into virus; self-inactivates the 5' LTR by a copying mechanism during viral genome integration; contains polyadenylation signal for transcription termination.
SV40 early pA	5955-6089	135	PolyA_signal	Simian virus 40 early polyadenylation signal	Allows transcription termination and polyadenylation of mRNA transcribed by Pol II RNA polymerase.
Ampicillin	7043-7903	861	ORF	Ampicillin resistance gene	Allows E. coli to be resistant to ampicillin.
pUC ori	8074-8662	589	rep_origin	pUC origin of replication	Facilitates plasmid replication in E. coli; regulates high-copy plasmid number (500-700).

Note: Components added by user are listed in **bold red** text.

Vector Sequence

```
  1 AATGTAGTCT TATGCAATAC TCTTGTAGTC TTGCAACATG GTAACGATGA GTTAGCAACA TGCCTTACAA GGAGAGAAAA AGCACCGTGC ATGCCGATTG
101 GTGGAAGTAA GGTGGTACGA TCGTGCCTTA TTAGGAAGGC AACAGACGGG TCTGACATGG ATTGGACGAA CCACTGAATT GCCGCATTGC AGAGATATTG
201 TATTTAAGTG CCTAGCTCGA TACATAAACG GGTCTCTCTG GTTAGACCAG ATCTGAGCCT GGGAGCTCTC TGGCTAACTA GGGAACCCAC TGCTTAAGCC
301 TCAATAAAGC TTGCCTTGAG TGCTTCAAGT AGTGTGTGCC CGTCTGTTGT GTGACTCTGG TAACTAGAGA TCCCTCAGAC CCTTTTAGTC AGTGTGGAAA
401 ATCTCTAGCA GTGGCGCCCG AACAGGGACT TGAAAGCGAA AGGGAAACCA GAGGAGCTCT CTCGACGCAG GACTCGGCTT GCTGAAGCGC GCACGGCAAG
```

501 AGGCGAGGGG CGGCGACTGG TGAGTACGCC AAAAATTTTG ACTAGCGGAG GCTAGAAGGA GAGAGATGGG TGCGAGAGCG TCAGTATTAA GCGGGGGAGA
601 ATTAGATCGC GATGGGAAAA AATTCGGTTA AGGCCAGGGG GAAAGAAAAA ATATAAATTA AAACATATAG TATGGGCAAG CAGGGAGCTA GAACGATTCG
701 CAGTTAATCC TGGCCTGTTA GAAACATCAG AAGGCTGTAG ACAAATACTG GGACAGCTAC AACCATCCCT TCAGACAGGA TCAGAAGAAC TTAGATCATT
801 ATATAATACA GTAGCAACCC TCTATTGTGT GCATCAAAGG ATAGAGATAA AAGACACCAA GGAAGCTTTA GACAAGATAG AGGAAGAGCA AAACAAAAGT
901 AAGACCACCG CACAGCAAGC GGCCGCTGAT CTTCAGACCT GGAGGAGGAG ATATGAGGGA CAATTGGAGA AGTGAATTAT ATAAATATAA AGTAGTAAAA
1001 ATTGAACCAT TAGGAGTAGC ACCCACCAAG GCAAAGAGAA GAGTGGTGCA GAGAGAAAAA AGAGCAGTGG GAATAGGAGC TTTGTTCCTT GGGTTCTTGG
1101 GAGCAGCAGG AAGCACTATG GGCGCAGCGT CAATGACGCT GACGGTACAG GCCAGACAAT TATTGTCTGG TATAGTGCAG CAGCAGAACA ATTTGCTGAG
1201 GGCTATTGAG GCGCAACAGC ATCTGTTGCA ACTCACAGTC TGGGGCATCA AGCAGCTCCA GGCAAGAATC CTGGCTGTGG AAAGATACCT AAAGGATCAA
1301 CAGCTCCTGG GGATTTGGGG TTGCTCTGGA AAACTCATTT GCACCACTGC TGTGCCTTGG AATGCTAGTT GGAGTAATAA ATCTCTGGAA CAGATTTGGA
1401 ATCACACGAC CTGGATGGAG TGGGACAGAG AAATTAACAA TTACACAAGC TTAATACACT CCTTAATTGA AGAATCGCAA AACCAGCAAG AAAAGAATGA
1501 ACAAGAATTA TTGGAATTAG ATAAATGGGC AAGTTTGTGG AATTGGTTTA ACATAACAAA TTGGCTGTGG TATATAAAAT TATTCATAAT GATAGTAGGA
1601 GGCTTGGTAG GTTTAAGAAT AGTTTTTGCT GTACTTTCTA TAGTGAATAG AGTTAGGCAG GGATATTCAC CATTATCGTT TCAGACCCAC CTCCCAACCC
1701 CGAGGGGACC CGACAGGCCC GAAGGAATAG AAGAAGAAGG TGGAGAGAGA GACAGAGACA GATCCATTCG ATTAGTGAAC GGATCTCGAC GGTATCGCTA
1801 GCTTTTAAAA GAAAAGGGGG GATTGGGGGG TACAGTGCAG GGGAAAGAAT AGTAGACATA ATAGCAACAG ACATACAAAC TAAAGAATTA CAAAAACAAA
1901 TTACAAAAAT TCAAAATTTT ACTAGTGATT ATCGGATCAA CTTTGTATAG AAAAGTTGGG CTCCGGTGCC CGTCAGTGGG CAGAGCGCAC ATCGCCCACA
2001 GTCCCCGAGA AGTTGGGGGG AGGGGTCGGC AATTGAACCG GTGCCTAGAG AAGGTGGCGC GGGGTAAACT GGGAAAGTGA TGTCGTGTAC TGGCTCCGCC
2101 TTTTTCCCGA GGGTGGGGGA GAACCGTATA TAAGTGCAGT AGTCGCCGTG AACGTTCTTT TTCGCAACGG GTTTGCCGCC AGAACACAGG TAAGTGCCGT
2201 GTGTGGTTCC CGCGGGCCTG GCCTCTTTAC GGGTTATGGC CCTTGCGTGC CTTGAATTAC TTCCACCTGG CTGCAGTACG TGATTCTTGA TCCCGAGCTT
2301 CGGGTTGGAA GTGGGTGGGA GAGTTCGAGG CCTTGCGCTT AAGGAGCCCC TTCGCCTCGT GCTTGAGTTG AGGCCTGGCC TGGGCGCTGG GGCCGCCGCG
2401 TGCGAATCTG GTGGCACCTT CGCGCCTGTC TCGCTGCTTT CGATAAGTCT CTAGCCATTT AAAATTTTTG ATGACCTGCT GCGACGCTTT TTTTCTGGCA
2501 AGATAGTCTT GTAAATGCGG GCCAAGATCT GCACACTGGT ATTTCGGTTT TTGGGGCCGC GGGCGGCGAC GGGGCCCGTG CGTCCCAGCG CACATGTTCG
2601 GCGAGGCGGG GCCTGCGAGC GCGGCCACCG AGAATCGGAC GGGGGTAGTC TCAAGCTGGC CGGCCTGCTC TGGTGCCTGG TCTCGCGCCG CCGTGTATCG
2701 CCCCGCCCTG GGCGGCAAGG CTGGCCCGGT CGGCACCAGT TGCGTGAGCG GAAAGATGGC CGCTTCCCGG CCCTGCTGCA GGGAGCTCAA AATGGAGGAC
2801 GCGGCGCTCG GGAGAGCGGG CGGGTGAGTC ACCCACACAA AGGAAAAGGG CCTTTCCGTC CTCAGCCGTC GCTTCATGTG ACTCCACGGA GTACCGGGCG
2901 CCGTCCAGGC ACCTCGATTA GTTCTCGAGC TTTTGGAGTA CGTCGTCTTT AGGTTGGGGG GAGGGGTTTT ATGCGATGGA GTTTCCCCAC ACTGAGTGGG
3001 TGGAGACTGA AGTTAGGCCA GCTTGGCACT TGATGTAATT CTCCTTGGAA TTTGCCCTTT TTGAGTTTGG ATCTTGGTTC ATTCTCAAGC CTCAGACAGT
3101 GGTTCAAAGT TTTTTTCTTC CATTTCAGGT GTCGTGACAA GTTTGTACAA AAAAGCAGGC TGCCACCATG GGCCCGCGCC GCCGGAGCCG AAAGCCCGAG
3201 GCCCCGAGGA GGCGCAGCCC GAGCCCGACC CCGACCCCCG GCCCCTCCCG GCGGGGCCCC TCCTTAGGCG CTTCCTCCCA TCAACACAGT CGGCGGAGAC
3301 AAGGTTGGCT AAAGGAGATC CGAAAGCTTC AGAAGAGCAC ACACCTCTTG ATAAGGAAGC TGCCCTTCAG CCGCCTGGCA AGAGAAATAT GTGTTAAATT
3401 CACTCGTGGT GTGGACTTCA ATTGGCAAGC CCAGGCCCTA TTGGCCCTAC AGAGGAGCAG AGAAGCATTT CTAGTTCATC TCTTTGAGGA CGCCTATCTC
3501 CTCACCTTAC ATGCAGGCCG AGTTACTCTC TTCCCAAAGG ATGTGCAACT GGCCCGGAGG ATCCGGGGCC TTGAGGAGGG ACTCGGCTGA ACCCAGCTTT
3601 CTTGTACAAA GTGGTGATAA TCGAATTCCG ATAATCAACC TCTGGATTAC AAAATTTGTG AAAGATTGAC TGGTATTCTT AACTATGTTG CTCCTTTTAC
3701 GCTATGTGGA TACGCTGCTT TAATGCCTTT GTATCATGCT ATTGCTTCCC GTATGGCTTT CATTTTCTCC TCCTTGTATA AATCCTGGTT GCTGTCTCTT
3801 TATGAGGAGT TGTGGCCCGT TGTCAGGCAA CGTGGCGTGG TGTGCACTGT GTTTGCTGAC GCAACCCCCA CTGGTTGGGG CATTGCCACC ACCTGTCAGC
3901 TCCTTTCCGG GACTTTCGCT TTCCCCCTCC CTATTGCCAC GGCGGAACTC ATCGCCGCCT GCCTTGCCCG CTGCTGGACA GGGGCTCGGC TGTTGGGCAC
4001 TGACAATTCC GTGGTGTTGT CGGGGAAGCT GACGTCCTTT CCATGGCTGC TCGCCTGTGT TGCCACCTGG ATTCTGCGCG GGACGTCCTT CTGCTACGTC
4101 CCTTCGGCCC TCAATCCAGC GGACCTTCCT TCCCGCGGCC TGCTGCCGGC TCTGCGGCCT CTTCCGCGTC TTCGCCTTCG CCCTCAGACG AGTCGGATCT
4201 CCCTTTGGGC CGCCTCCCCG CATCGGGAAT TCCCGCGGTT CGAACGCGTT GACATTGATT ATTGACTAGT TATTAATAGT AATCAATTAC GGGGTCATTA
4301 GTTCATAGCC CATATATGGA GTTCCGCGTT ACATAACTTA CGGTAAATGG CCCGCCTGGC TGACCGCCCA ACGACCCCCG CCCATTGACG TCAATAATGA
4401 CGTATGTTCC CATAGTAACG CCAATAGGGA CTTTCCATTG ACGTCAATGG GTGGAGTATT TACGGTAAAC TGCCCACTTG GCAGTACATC AAGTGTATCA
4501 TATGCCAAGT ACGCCCCCTA TTGACGTCAA TGACGGTAAA TGGCCCGCCT GGCATTATGC CCAGTACATG ACCTTATGGG ACTTTCCTAC TTGGCAGTAC
4601 ATCTACGTAT TAGTCATCGC TATTACCATG GTGATGCGGT TTTGGCAGTA CATCAATGGG CGTGGATAGC GGTTTGACTC ACGGGGATTT CCAAGTCTCC
4701 ACCCCATTGA CGTCAATGGG AGTTTGTTTT GGCACCAAAA TCAACGGGAC TTTCCAAAAT GTCGTAACAA CTCCGCCCCA TTGACGCAAA TGGGCGGTAG
4801 GCGTGTACGG TGGGAGGTCT ATATAAGCAG AGCTCTCTGG CTAACTAGAG AACCCACTGC GCCACCATGG TGAGCAAGGG CGAGGAGGAT AACATGGCCA
4901 TCATCAAGGA GTTCATGCGC TTCAAGGTGC ACATGGAGGG CTCCGTGAAC GGCCACGAGT TCGAGATCGA GGGCGAGGGC GAGGGCCGCC CCTACGAGGG
5001 CACCCAGACC GCCAAGCTGA AGGTGACCAA GGGTGGCCCC CTGCCCTTCG CCTGGGACAT CCTGTCCCCT CAGTTCATGT ACGGCTCCAA GGCCTACGTG
5101 AAGCACCCCG CCGACATCCC CGACTACTTG AAGCTGTCCT TCCCCGAGGG CTTCAAGTGG GAGCGCGTGA TGAACTTCGA GGACGGCGGC GTGGTGACCG
5201 TGACCCAGGA CTCCTCCCTG CAGGACGGCG AGTTCATCTA CAAGGTGAAG CTGCGCGGCA CCAACTTCCC CTCCGACGGC CCCGTAATGC AGAAGAAGAC
5301 CATGGGCTGG GAGGCCTCCT CCGAGCGGAT GTACCCCGAG GACGGCGCCC TGAAGGGCGA GATCAAGCAG AGGCTGAAGC TGAAGGACGG CGGCCACTAC
5401 GACGCTGAGG TCAAGACCAC CTACAAGGCC AAGAAGCCCG TGCAGCTGCC CGGCGCCTAC AACGTCAACA TCAAGTTGGA CATCACCTCC CACAACGAGG
5501 ACTACACCAT CGTGGAACAG TACGAACGCG CCGAGGGCCG CCACTCCACC GGCGGCATGG ACGAGCTGTA CAAGTAAGGT ACCTTTAAGA CCAATGACTT
5601 ACAAGGCAGC TGTAGATCTT AGCCACTTTT TAAAAGAAAA GGGGGGACTG GAAGGGCTAA TTCACTCCCA ACGAAGACAA GATCTGCTTT TTGCTTGTAC
5701 TGGGTCTCTC TGGTTAGACC AGATCTGAGC CTGGGAGCTC TCTGGCTAAC TAGGGAACCC ACTGCTTAAG CCTCAATAAA GCTTGCCTTG AGTGCTTCAA
5801 GTAGTGTGTG CCCGTCTGTT GTGTGACTCT GGTAACTAGA GATCCCTCAG ACCCTTTTAG TCAGTGTGGA AAATCTCTAG CAGTAGTAGT TCATGTCATC
5901 TTATTATTCA GTATTTATAA CTTGCAAAGA AATGAATATC AGAGAGTGAG AGGAACTTGT TTATTGCAGC TTATAATGGT TACAAATAAA GCAATAGCAT
6001 CACAAATTTC ACAAATAAAG CATTTTTTTC ACTGCATTCT AGTTGTGGTT TGTCCAAACT CATCAATGTA TCTTATCATG TCTGGCTCTA GCTATCCCGC
6101 CCCTAACTCC GCCCATCCCG CCCCTAACTC CGCCCAGTTC CGCCCATTCT CCGCCCCATG GCTGACTAAT TTTTTTTATT TATGCAGAGG CCGAGGCCGC
6201 CTCGGCCTCT GAGCTATTCC AGAAGTAGTG AGGAGGCTTT TTTGGAGGCC TAGGGACGTA CCCAATTCGC CCTATAGTGA GTCGTATTAC GCGCGCTCAC
6301 TGGCCGTCGT TTTACAACGT CGTGACTGGG AAAACCCTGG CGTTACCCAA CTTAATCGCC TTGCAGCACA TCCCCCTTTC GCCAGCTGGC GTAATAGCGA
6401 AGAGGCCCGC ACCGATCGCC CTTCCCAACA GTTGCGCAGC CTGAATGGCG AATGGGACGC GCCCTGTAGC GGCGCATTAA GCGCGGCGGG TGTGGTGGTT
6501 ACGCGCAGCG TGACCGCTAC ACTTGCCAGC GCCCTAGCGC CCGCTCCTTT CGCTTTCTTC CCTTCCTTTC TCGCCACGTT CGCCGGCTTT CCCCGTCAAG
6601 CTCTAAATCG GGGGCTCCCT TTAGGGTTCC GATTTAGTGC TTTACGGCAC CTCGACCCCA AAAAACTTGA TTAGGGTGAT GGTTCACGTA GTGGGCCATC
6701 GCCCTGATAG ACGGTTTTTC GCCCTTTGAC GTTGGAGTCC ACGTTCTTTA ATAGTGGACT CTTGTTCCAA ACTGGAACAA CACTCAACCC TATCTCGGTC
6801 TATTCTTTTG ATTTATAAGG GATTTTGCCG ATTTCGGCCT ATTGGTTAAA AAATGAGCTG ATTTAACAAA AATTTAACGC GAATTTTAAC AAAATATTAA
6901 CGCTTACAAT TTAGGTGGCA CTTTTCGGGG AAATGTGCGC GGAACCCCTA TTTGTTTATT TTTCTAAATA CATTCAAATA TGTATCCGCT CATGAGACAA
7001 TAACCCTGAT AAATGCTTCA ATAATATTGA AAAAGGAAGA GTATGAGTAT TCAACATTTC CGTGTCGCCC TTATTCCCTT TTTTGCGGCA TTTTGCCTTC
7101 CTGTTTTTGC TCACCCAGAA ACGCTGGTGA AAGTAAAAGA TGCTGAAGAT CAGTTGGGTG CACGAGTGGG TTACATCGAA CTGGATCTCA ACAGCGGTAA
7201 GATCCTTGAG AGTTTTCGCC CCGAAGAACG TTTTCCAATG ATGAGCACTT TTAAAGTTCT GCTATGTGGC GCGGTATTAT CCCGTATTGA CGCCGGGCAA
7301 GAGCAACTCG GTCGCCGCAT ACACTATTCT CAGAATGACT TGGTTGAGTA CTCACCAGTC ACAGAAAAGC ATCTTACGGA TGGCATGACA GTAAGAGAAT
7401 TATGCAGTGC TGCCATAACC ATGAGTGATA ACACTGCGGC CAACTTACTT CTGACAACGA TCGGAGGACC GAAGGAGCTA ACCGCTTTTT TGCACAACAT
7501 GGGGGATCAT GTAACTCGCC TTGATCGTTG GGAACCGGAG CTGAATGAAG CCATACCAAA CGACGAGCGT GACACCACGA TGCCTGTAGC AATGGCAACA
7601 ACGTTGCGCA AACTATTAAC TGGCGAACTA CTTACTCTAG CTTCCCGGCA ACAATTAATA GACTGGATGG AGGCGGATAA AGTTGCAGGA CCACTTCTGC
7701 GCTCGGCCCT TCCGGCTGGC TGGTTTATTG CTGATAAATC TGGAGCCGGT GAGCGTGGGT CTCGCGGTAT CATTGCAGCA CTGGGGCCAG ATGGTAAGCC
7801 CTCCCGTATC GTAGTTATCT ACACGACGGG GAGTCAGGCA ACTATGGATG AACGAAATAG ACAGATCGCT GAGATAGGTG CCTCACTGAT TAAGCATTGG
7901 TAACTGTCAG ACCAAGTTTA CTCATATATA CTTTAGATTG ATTTAAAACT TCATTTTTAA TTTAAAAGGA TCTAGGTGAA GATCCTTTTT GATAATCTCA
8001 TGACCAAAAT CCCTTAACGT GAGTTTTCGT TCCACTGAGC GTCAGACCCC GTAGAAAAGA TCAAAGGATC TTCTTGAGAT CCTTTTTTTC TGCGCGTAAT
8101 CTGCTGCTTG CAAACAAAAA AACCACCGCT ACCAGCGGTG GTTTGTTTGC CGGATCAAGA GCTACCAACT CTTTTTCCGA AGGTAACTGG CTTCAGCAGA
8201 GCGCAGATAC CAAATACTGT TCTTCTAGTG TAGCCGTAGT TAGGCCACCA CTTCAAGAAC TCTGTAGCAC CGCCTACATA CCTCGCTCTG CTAATCCTGT
8301 TACCAGTGGC TGCTGCCAGT GGCGATAAGT CGTGTCTTAC CGGGTTGGAC TCAAGACGAT AGTTACCGGA TAAGGCGCAG CGGTCGGGCT GAACGGGGGG
8401 TTCGTGCACA CAGCCCAGCT TGGAGCGAAC GACCTACACC GAACTGAGAT ACCTACAGCG TGAGCTATGA GAAAGCGCCA CGCTTCCCGA AGAGAAAGG
8501 CGGACAGGT ATCCGGTAAG CGGCAGGGTC GGAACAGGAG AGCGCACGAG GGAGCTTCCA GGGGGAAACG CCTGGTATCT TTATAGTCCT GTCGGGTTTC
8601 GCCACCTCTG ACTTGAGCGT CGATTTTTGT GATGCTCGTC AGGGGGGCGG AGCCTATGGA AAAACGCCAG CAACGCGGCC TTTTTACGGT TCCTGGCCTT
8701 TTGCTGGCCT TTTGCTCACA TGTTCTTTCC TGCGTTATCC CCTGATTCTG TGGATAACCG TATTACCGCC TTTGAGTGAG CTGATACCGC TCGCCGCAGC
8801 CGAACGACCG AGCGCAGCGA GTCAGTGAGC GAGGAAGCGG AAGAGCGCCC AATACGCAAA CCGCCTCTCC CCGCGCGTTG GCCGATTCAT TAATGCAGCT

```
8901  GGCACGACAG GTTTCCCGAC TGGAAAGCGG GCAGTGAGCG CAACGCAATT AATGTGAGTT AGCTCACTCA TTAGGCACCC CAGGCTTTAC ACTTTATGCT
9001  TCCGGCTCGT ATGTTGTGTG GAATTGTGAG CGGATAACAA TTTCACACAG GAAACAGCTA TGACCATGAT TACGCCAAGC GCGCAATTAA CCCTCACTAA
9101  AGGGAACAAA AGCTGGAGCT GCAAGCTT
```

Validation by Restriction Enzyme Digestion

Cutters	Locations	Fragments (bp)
**XhoI**	2925	9128
**AgeI**	2038	9128
**SpeI**	1922, 4266	2344, 6784
**ApaLI**	3843, 4928, 7159, 8405	1085, 2231, 1246, 4566
**DraIII**	2996, 6691	3695, 5433
**ApaLI+XhoI**	2925, 3843, 4928, 7159, 8405	918, 1085, 2231, 1246, 3648
**ApaLI+AgeI**	2038, 3843, 4928, 7159, 8405	1805, 1085, 2231, 1246, 2761
**ApaLI+SpeI**	1922, 3843, 4266, 4928, 7159, 8405	1921, 423, 662, 2231, 1246, 2645
**ApaLI+DraIII**	2996, 3843, 4928, 6691, 7159, 8405	847, 1085, 1763, 468, 1246, 3719
